# Reconstructing the complex evolutionary history of mobile plasmids in red algal genomes

**DOI:** 10.1038/srep23744

**Published:** 2016-03-31

**Authors:** JunMo Lee, Kyeong Mi Kim, Eun Chan Yang, Kathy Ann Miller, Sung Min Boo, Debashish Bhattacharya, Hwan Su Yoon

**Affiliations:** 1 Department of Biological Sciences, Sungkyunkwan University, Suwon, 16419, Korea; 2 Marine Biodiversity Institute of Korea, Seocheon, 325-902, Korea; 3 Marine Ecosystem Research Division, Korea Institute of Ocean Science & Technology, Ansan, 15627, Korea; 4 Herbarium, University of California at Berkeley, 1001 Valley Life Sciences Building 2465, Berkeley, California, 94720-2465, USA; 5 Department of Biology, Chungnam National University, Daejeon, 34134, Korea; 6 Department of Ecology, Evolution and Natural Resources and Department of Marine and Coastal Sciences, Rutgers University, New Brunswick, NJ 08901, USA

## Abstract

The integration of foreign DNA into algal and plant plastid genomes is a rare event, with only a few known examples of horizontal gene transfer (HGT). Plasmids, which are well-studied drivers of HGT in prokaryotes, have been reported previously in red algae (Rhodophyta). However, the distribution of these mobile DNA elements and their sites of integration into the plastid (ptDNA), mitochondrial (mtDNA), and nuclear genomes of Rhodophyta remain unknown. Here we reconstructed the complex evolutionary history of plasmid-derived DNAs in red algae. Comparative analysis of 21 rhodophyte ptDNAs, including new genome data for 5 species, turned up 22 plasmid-derived open reading frames (ORFs) that showed syntenic and copy number variation among species, but were conserved within different individuals in three lineages. Several plasmid-derived homologs were found not only in ptDNA but also in mtDNA and in the nuclear genome of green plants, stramenopiles, and rhizarians. Phylogenetic and plasmid-derived ORF analyses showed that the majority of plasmid DNAs originated within red algae, whereas others were derived from cyanobacteria, other bacteria, and viruses. Our results elucidate the evolution of plasmid DNAs in red algae and suggest that they spread as parasitic genetic elements. This hypothesis is consistent with their sporadic distribution within Rhodophyta.

Horizontal gene transfer (HGT) plays a significant role in the evolution of bacterial genomes, promoting environmental adaptation and speciation. Plasmids drive HGT by moving DNA from one genome to another, often between species, in the absence of sexual reproduction^1^,^2^,^3^. However, the mechanism of eukaryotic HGT is poorly understood, although it is known to occur from prokaryotes to eukaryotes^4^,^5^,^6^,^7^, between different eukaryotes^8^,^9^,^10^, and from eukaryotes to prokaryotes^11^,^12^. A special case of HGT, endosymbiotic gene transfer (EGT), is responsible for massive amounts of intracellular gene movement in eukaryotes. This is an outcome of organellogenesis, whereby 100 s to 1000 s of genes were transferred from the bacterium-derived organelle genomes (i.e., the mitochondrion and plastid) to the host nuclear chromosomes^13^,^14^,^15^,^16^.

Nuclear-encoded, plastid-derived genes have been studied in the glaucophyte alga *Cyanophora paradoxa* Korshikov (6–11%)^17^,^18^; the red alga *Cyanidioschyzon merolae* DeLuca, Taddei, & Varano (6–20%)^19^,^20^,^21^; the green algae *Chlamydomonas reinhardtii* P.C.A. Dangeard (6–14%) and *Ostreococcus tauri* C. Courties & M.-J. Chrétiennot-Dinet (11%)^20^,^21^,^22^; and in *Arabidopsis thaliana* (L.) Heynh. and other land plants (9–18%)^20^,^21^,^23^. EGT is essentially uni-directional. As a consequence, organelle (e.g., plastid) genomes have been reduced to 100 ~200 Kbp from their original size of several megabases in the cyanobacterial endosymbiont. It is not known, however, if plasmids may have facilitated EGT in algae and plants, thereby contributing significantly to their genome reduction.

HGTs have also been reported between organelle genomes of unrelated organisms. The plastid genome of the common milkweed *Asclepias syriaca* L. contains several mitochondrial genes^24^, whereas the mitochondrial genome of *Amborella trichopoda* Baill. contains mtDNAs from green algae (including the entire mitochondrial genome in three species), mosses, and other angiosperms^25^,^26^,^27^. The maize mitochondrial S-1 plasmid was found in the mitochondrial genome of the liverwort *Marchantia polymorpha* L.^28^,^29^. Interestingly, these sequences are similar to the mitochondrial *dpo* gene in the red alga *Porphyra* and the golden-brown alga *Ochromonas danica* E. Pringsheim^30^, suggesting the existence of HGT among different phyla. Plasmid-derived sequences were also reported from mtDNA in the brown alga *Pylaiella littoralis* (L.) Kjellman^31^ and two fungal species, *Agaricus bisporus* (J.E. Lange) Imbach and *Gigaspora rosea* T.H. Nicolson & N.C. Schenck^32^,^33^. The plastid genomes of photosynthetic haptophytes and cryptophytes contain bacterial-derived *rpl*36 genes^7^, and the cryptophyte *Rhodomonas salina* D.R.A. Hill & R. Wetherbee has a bacterial *dna*X gene in its ptDNA^34^. These examples demonstrated significant HGT between organelles and between organelles and plasmids; however, no such data has been reported for the red algae (Rhodophyta).

In red algae, proteobacterial operons related to leucine biosynthesis (*leuC* and *leuD* subunits) are encoded in the plastid genome of *Gracilaria tenuistipitata* var. *liui* J. Zhang, & B. Xia^35^,^36^. This gene cluster (*leu*A/B/C/D) was traced to a plasmid from *Buchnera*, a genus of bacterial endosymbionts in aphids^37^,^38^. The ptDNA of the red alga *Pyropia haitanensis* (T. J. Chang & B.F. Zheng) N. Kikuchi & M. Miyata contains plasmid-derived sequences that were discovered in the plasmid of another red alga, *Porphyra pulchra* G.J. Hollenberg^39^,^40^. The mtDNA of other red algae, *Gracilaria chilensis* C.J. Bird, J.L. McLachlan, & E.C. de Oliveira*, Gracilariopsis chorda* (E.M. Holmes) Ohmi and *Gracilariopsis lemaneiformis* (Bory de Saint-Vincent) Dawson, Acleto, & Foldvik, contain partial plasmid sequences that have been reported in *Gracilaria robusta* Setchell^41^,^42^,^43^. However, less is known about the mechanisms of plasmid-derived HGT to the plastid genome.

Plasmids are extrachromosomal genetic materials that are generally referred to as autonomously replicating double-stranded, circular or linear DNA molecules^44^. About 25% of red algal genera contain more than two plasmids per species, and encode open reading frames (ORFs) that are transcriptionally active^45^. Eukaryotic plasmids are widely distributed throughout algae, land plants, fungi, yeast, and other eukaryotes. However, their origins are poorly understood and their functions, including pathogenicity, have been reported only in a few cases^45^,^46^,^47^,^48^. Of 35 red algal species assessed for plasmid sequences, 5 species contain 14 plasmid sequences: *Porphyra pulchra* (five plasmids); *Pyropia tenera* (Kjellman) N. Kikuchi, M. Miyata, M.S. Hwang & H.G. Choi (two plasmids); *Gracilaria chilensis* (three plasmids); *G. robusta* (two plasmids); and *Gracilariopsis lemaneiformis* (two plasmids)^39^,^45^,^49^,^50^,^51^,^52^. However, no comprehensive analysis has yet been done to investigate the evolutionary relationship between plasmid DNA and plastid genomes.

To this end, we sequenced five red algal ptDNAs, including two that are plasmid-rich from *Gracilaria chilensis* and *Porphyra pulchra.* We analyzed plasmid-derived sequences from a total of 21 available red algal plastid genomes^35^,^36^,^40^,^53^,^54^,^55^,^56^,^57^,^58^,^59^,^60^,^61^ to elucidate the impact of plasmids over the >1 billion year evolutionary history of red algae.

## Results and discussion

### Novel red algal plastid genomes

Five novel plastid genomes were completed using next-generation sequencing (NGS) data from *Gelidium elegans* (1,529 Mbp of total data), *G. vagum* (990 Mbp), *Gracilaria chilensis* (506 Mbp), *Porphyra pulchra* (263 Mbp) and *Sporolithon durum* (3,190 Mbp). The range of average genome coverage from the raw data was 52 ~445x (Supplementary Table S1). The plastid genome of *P. pulchra* (Supplementary Fig. S1) was the largest (194,175 bp) and had a higher GC-content (33.3%) than that of *S. durum* (191,465 bp, GC = 29.3%, Supplementary Fig. S2), *G. elegans* (174,748 bp, GC = 30.2%, Supplementary Fig. S3), *G. vagum* (179,853 bp, GC = 29.9%, Supplementary Fig. S4) and *Gr. chilensis* (185,637 bp, GC = 29.3%, Supplementary Fig. S5). Basic information about these plastid genomes is summarized in Supplementary Table S2. The plastid genome of *P. pulchra,* similar to those in other bangiophycean species, comprised 207 protein-coding genes, 37 tRNAs and 6 rRNAs; the rRNA operon (*rrs*, *rrl* and *rrf*) was duplicated. Among the florideophycean species, *S. durum* comprised 202 protein-coding genes, 30 tRNAs, 3 rRNAs, 3 rRNAs and 2 introns, as well as several pseudogenes (*dna*B, *syf*B, *ycf*21 and *ycf*23). This genome lacked the *syh* gene and *trn*V tRNA, both of which are present in another member of the Corallinophycidae, *Calliarthron tuberculosum*. These two coralline algae have a unique group II intron in the *chl*B gene^36^ with intronic *orfs. Gelidium vagum* contained 201 protein-coding genes with pseudogenes of *ycf*34; *G. elegans* contained 202 protein-coding genes. These members of the order Gelidiales encode 30 tRNAs, 3 rRNAs and a group II intron in *trn*Me tRNA^36^. The plastid genome of *Gracilaria chilensis* (order Gracilariales) contained 203 protein-coding genes, 30 tRNAs, 3 rRNAs and a group II intron in *trn*Me tRNA, which had not been found previously in the plastid genomes of *G. salicornia* and *G. tenuistipitata* var. *liui* or in *Grateloupia taiwanensis* (Halymeniales).

**Figure 1:**
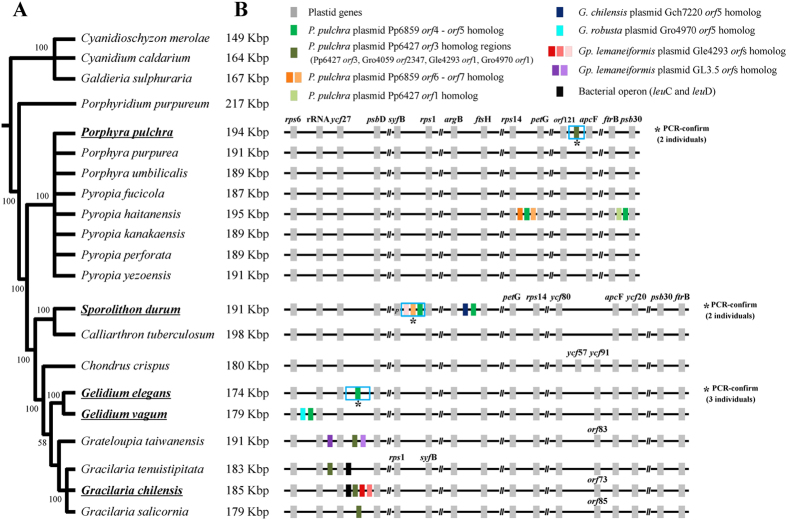
Phylogeny of red algae showing the distribution of plasmid-derived DNA. (**A**) Simplified maximum likelihood (ML) tree topology for red algae based on concatenated 193 protein encoding genes of plastid genomes (see also Supplementary Fig. S6). (**B**) The plastid genome sizes (kilo base pair, Kbp) are shown beside the taxon names. Colored boxes indicate plastid genes (grey), plasmid-derived regions: *Porphyra pulchra* plasmid Pp6859 *orf*4 and *orf*5 homolog (green), *P. pulchra* plasmid Pp6427 *orf*3 homolog (dark green), *P. pulchra* plasmid Pp6859 *orf*6 and *orf*7 (orange and bright orange), *P. pulchra* plasmid Pp6427 *orf*1 homolog (bright green), *Gracilaria chilensis* plasmid Gch7220 *orf*5 homolog (blue), *G. robusta* plasmid Gro4970 *orf*5 homolog (cyan), *Gracilariopsis lemaneiformis* plasmid Gle4293 *orf*s homolog (red, pink and bright pink), *Gp. lemaneiformis* plasmid GL3.5 *orf*s homolog (violet and bright violet), and genes that encode the bacterial operon for *leu*C and *leu*D (black). Detailed information about plasmid-derived regions is given in Supplementary Fig. S7. The “*p*” in *syf*B gene of *Sporolithon durum* designates a pseudogenization of the gene. PCR-confirmed regions in different individuals are marked by asterisk. Syntenic diagrams for the *Cyanidioschyzon*, *Cyanidium*, *Galdieria* and *Porphyridium* are not shown because there was no plasmid-derived DNA.

The ML tree inferred from the concatenated dataset of 193 plastid protein-coding genes (Supplementary Table S3; Table S4) resolved phylogenetic relationships among red algae (Fig. 1A, Supplementary Fig. S6). The early diverging Cyanidiophyceae was chosen as the outgroup for this phylogeny^62^,^63^. The Bangiophyceae and the Florideophyceae grouped together with maximum ML bootstrap support value (MLB, 100%), and each class formed a strongly supported monophyletic clade, as previously reported^62^,^63^,^64^,^65^. Within the Bangiophyceae, *Porphyra pulchra* grouped within *Pyropia* clade (100% MLB) rather than *Porphyra* clade, suggesting a taxonomic revision of *Porphyra pulchra* as *Pyropia pulchra*. Relationships within the Florideophyceae were consistent with previous work^64^,^65^,^66^. For example, two Corallinophycidae species, *Sporolithon durum* (Sporolithales) and *Calliarthron tuberculosum* (Corallinales) grouped together (100% MLB) and were sister to the rest of florideophycean clades. Within the subclass Rhodymeniophycidae, *Chondrus crispus* (Gigartinales) diverged first, followed by *Gelidium* (Gelidiales), *Grateloupia taiwanensis* (Halymeniales) and *Gracilaria* (Gracilariales). Although internal relationships within the Rhodymeniophycidae were not resolved with the concatenated plastid dataset, we used this ML tree (Fig. 1A) as a reference for inferring the evolution of red algal plasmid DNAs.

### Distribution of plasmid-derived genes in red algal ptDNA

We identified 22 plasmid-derived (PD) sequences in nine red algal species when 56 red algal plasmid-encoded proteins were used to query the available 21 red algal plastid genomes (using BLASTx, *e*-value ≤ 1.0*e*^−05^) (GI numbers of the 56 proteins are listed in Table 1). The putative origin, copy number, and distribution in the ptDNAs were different for each ORF (Fig. 1B; Supplementary Table S5). In addition to the previously reported bacterial operon *leu*C and *leu*D gene^35^,^36^ (two black blocks in Fig. 1B), out of the 22 PD *orf*s (including pseudogenized regions) identified here, six were homologous to *orf*4 and *orf*5 of the *Porphyra pulchra* plasmid Pp6859 (GI: 11466614; green region in Fig. 1B), five were homologous to the *P. pulchra* plasmid Pp6427 (GI: 11466608) *orf*3 (dark green region in Fig. 1B), and two were homologous to the *P. pulchra* plasmid Pp6859 *orf*6 (bright orange region in Fig. 1B). The rest of the PD *orfs* were unique to plasmids in their species of origin. Interestingly, six homologous PD sequences from Pp6859 *orf*4 and *orf*5 (green box in Fig. 1B) were found in four red algal plastid genomes but their copy number and position were not consistent with their phylogenetic relationships. For instance, two copies of the Pp6859 *orf*4-*orf*5 homolog were found in *Pyropia haitanensis* among eight *Porphyra/Pyropia* species, whereas a single copy was found in each *Gelidium* species, but at different locations. *Sporolithon durum* contained two homologous copies but one was pseudogenized. The sequences homologous to plasmid Pp6427 *orf*3 of *P. pulchra*^39^ were found in the plastid genomes of three *Gracilaria* species and *Grateloupia taiwanensis* (dark-green in Fig. 1B) in addition to that of *P. pulchra*, and were located near ribosomal RNAs and *ycf*27 genes. We note that half of the PD *orf*s were positioned near rRNA (*rps*6-rRNA-*ycf*27-*psb*D, see Fig. 1B), in particular in *Gelidium, Grateloupia,* and *Gracilaria*.

We tested whether these PD *orf*s were conserved in populations within a species and in different individuals within a population. To this end, PCR was used to test three populations of *G. elegans* (SKKU18, SKKU22, SKKU28), two individuals of *P. pulchra* selected from a single population (UC1879714 and UC1454976), and three individuals of *S. durum* from a single population (SKKU_SD01, SKKU_SD02, and SKKU_SD03; Supplementary Table S6). All tested PD *orfs* were found in the same position with the same flanking region sequences. Therefore, these PD *orfs* are conserved across different individuals within one species.

For *P. pulchra* and *G. chilensis*, the PD *orf*s found in their ptDNA or their homologs were not detected in the draft genome data but rather only in the plasmid sequence. From draft genome data (NGS), five complete plasmid sequences (103 ~360× average coverage) were recovered from *Porphyra pulchra* (263 Mbp of reads) and three plasmids (401 ~590× average coverage) from *Gracilaria chilensis* (506 Mbp of reads). Thus, NGS data were useful for identifying plasmid sequences. However, we could not find red algal plasmid sequences in the published complete genome of *Cyanidioschyzon merolae*^67^,^68^, *Galdieria sulphuraria*^69^, *Porphyridium purpureum*^70^, *Calliarthron tuberculosum*^71^, *Chondrus crispus*^56^, and *Gelidium vagum* (Yoon *et al.* unpublished).

### Origin of the plasmid-derived Pp6859 *orf*4-*orf*5 homologs in ptDNA

**Figure 2:**
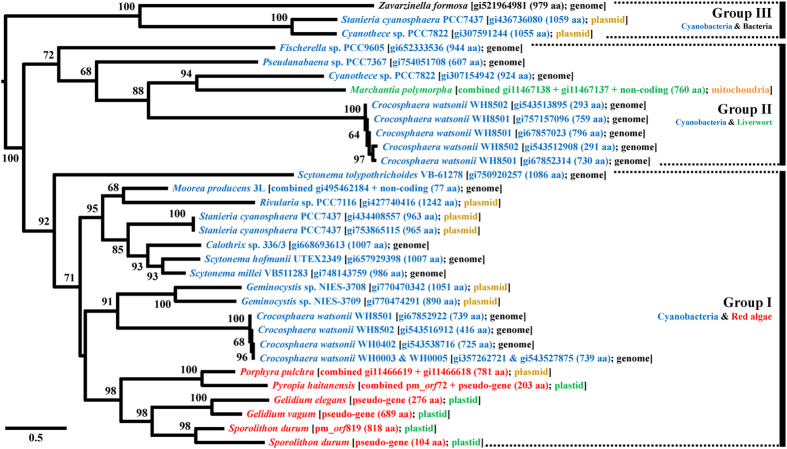
Maximum likelihood (ML) tree based on aligned amino acid sequences of homologous regions of *Porphyra pulchra* plasmid Pp6859 *orf*4 and *orf*5 with 2,000 ML bootstrap replications. Species names are followed by GI, amino acid (aa) length, and location. Colored names indicate cyanobacteria (cyan), bacteria (black), liverwort (bright green) and red algae (red). Locations of the sequences are genome (black), plastid (green), mitochondria (orange) and plasmid (yellowish brown). Some *orf*s and pseudogenized or non-coding regions were combined and aligned with sampled taxon sequences (Supplementary Table S3; Table S4; Table S7). The clades of the ML tree are divided into three groups based on species composition. Group I includes cyanobacterial plasmids and genomes with red algal plasmid and plastid regions. Group II includes cyanobacterial genomes and mitochondrial regions of liverwort, *Marchantia polymorpha*). Group III includes cyanobacterial plasmids and a bacterial (*Zavarzinella formosa*) genome.

**Figure 3:**
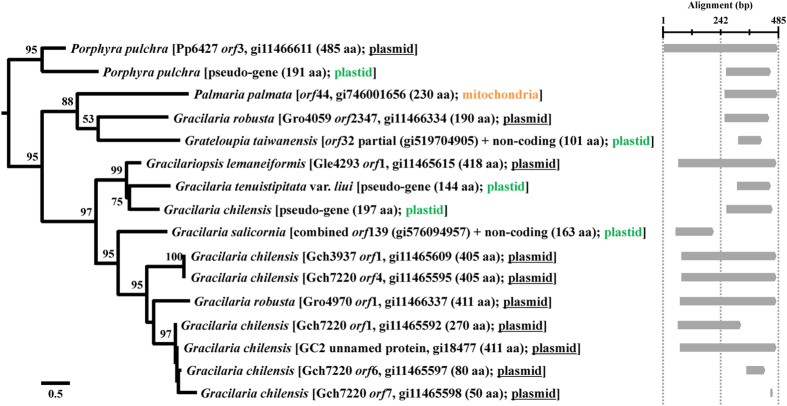
Maximum likelihood (ML) tree based on aligned amino acid sequences of homologous regions to *Porphyra pulchra* plasmid Pp6427 *orf*3 with 2,000 ML bootstrap replications. Species names are followed by source, GI, amino acid (aa) length, and location. Some *orf*s and pseudogenized or non-coding regions were combined and aligned with sampled taxon sequences (Supplementary Table S7). Location of sequences is indicated by color: plasmid (underlined black), plastid (green) and mitochondria (orange). Synteny is shown with the schematic alignment on the right of the tree based on major regions of homology.

**Figure 4:**
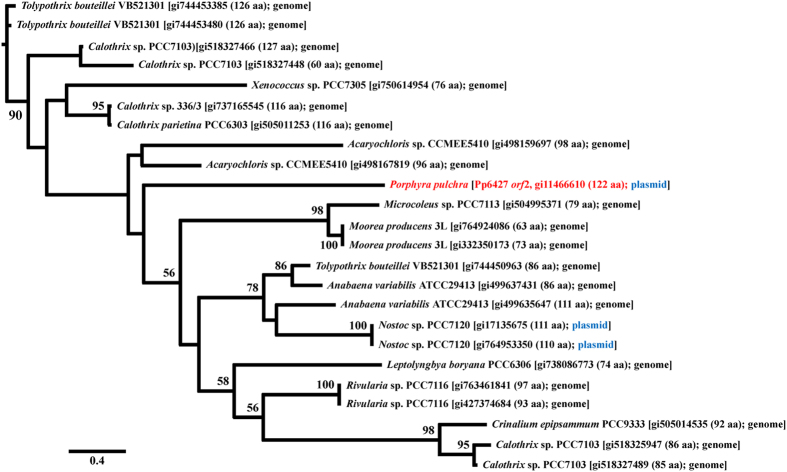
Maximum likelihood (ML) tree based on aligned amino acid sequences of homologous genes of *Porphyra pulchra* plasmid Pp6427 *orf*2 (red) with 2,000 ML bootstrap replications. Blue indicates that the sequences found in the plasmid genome.

The origin of plasmid-derived *orf*s was difficult to determine because most plastid-encoded PD *orf*s matched only plasmid *orf* data, except for the following five cases (see, Figs 2, 3, 4, S8, S9). A BLAST search against the NCBI database using six homologous plastid genes of the *P. pulchra* plasmid Pp6859 *orf*4-*orf*5 resulted in 26 hypothetical proteins encoded in a bacterial genome, cyanobacterial genomes, cyanobacterial plasmids, and the mitochondrial genome of a liverwort. All homologous sequences of Pp6859 *orf*4-*orf*5 were used to reconstruct the ML phylogeny using RAxML (Fig. 2). In the best tree, red algal plastid PD *orf*s grouped together, including plasmid Pp6859 (98% MLB). It is interesting that plasmid genes of Pp6859 (*P. pulchra*) grouped with pseudogenized plastid genes from *P. haitanensis* (100% MLB), suggesting a possible ORF gene transfer mediated by a plasmid to a plastid genome (see discussion in previous study^40^).

The red algal clade was positioned within cyanobacterial clade Group I (92% MLB) that included hypothetical proteins encoded in the cyanobacterial genome as well as cyanobacterial plasmids (Fig. 2). Group II (72% MLB) contained cyanobacterial species and mitochondrial sequences from the liverwort *Marchantia polymorpha* (combined with two fragmented genes with flanking region data). Moon and Goff^39^ reported the putative homologous relationship between Pp6859 and the liverwort mitochondrial region. Two cyanobacterial plasmid genes and a hypothetical gene from the Planctomycetes *Zavarzinella* f*ormosa* were grouped together (Group III, 100% MLB).

Because only 12 species (16 strains; Fig. 2) out of the 100 cyanobacterial genomes available in NCBI contain a homolog of Pp6859 *orf*4-*orf*5, it is unlikely to be a core cyanobacterial gene. If this *orf* was inherited from the primary endosymbiosis event, it should be retained in most red algal plastid genomes as well as those of other primary endosymbiotic lineages (i.e., green and glaucophyte algae). However, it is sporadically distributed in only a few species (e.g., *Pyropia*, *Gelidium* and *Sporolithon*) (Fig. 1). We postulate that this *orf* originated from an unknown cyanobacterial species, then spread independently to other cyanobacteria, to a bacterium (*Z. formosa*), to a liverwort (*M. polymorpha*), and to a few red algae.

The cyanobacterium *Crocosphaera watsonii* WH8501 contains three copies of this *orf* as a result of gene duplications^72^,^73^. However, it is likely that these red algal PD *orf*s originated independently, as a result of plasmid mobility. Alternatively, a red algal species inherited this *orf* from a cyanobacterial genome through the plasmid, after which it was transferred into the plastid genome in random genomic positions (e.g., see Fig. 1B), followed by pseudogenization or complete loss. This plasmid-mediated HGT may have occurred after speciation. For example, two *Gelidium* species both retain PD *orf*s, but they differ in size and genomic position. Similar cases were found in three *Gracilaria* species. If indeed the PD *orf*s were introduced during speciation, the presence and position of PD *orf*s could be used as species-specific markers.

### Origin of the plasmid-derived Pp6427 *orf*s homologs in plastid genomes

Pp6427 *orf*3 homologs were found in five plastid regions (Fig. 1), nine plasmid *orf*s and a mitochondrial *orf* from seven red algal species. Unlike Pp6859, homologous sequences were not found in any other taxa; therefore, Pp6427 *orf*3 homologs are specific to red algae. In the ML tree using 16 homologs (Fig. 3), strong (>95% MLB) plasmid-plastid relationships were recovered, even though all plastid-encoded *orfs* were pseudogenized (see alignment in Fig. 3). For example, the plasmid Pp6427 o*rf*3 (485 aa) grouped with a short pseudogenized gene (191 aa) in the plastid genome of *P. pulchra* (95% MLB), whereas the plasmid Gro4059 (GI: 11466333) *orf*2347 (190 aa) grouped with partial genes from *G. taiwanensis* (101 aa) within a clade of mitochondrial *orf*44 from *Palmaria palmata* (Linnaeus) F. Weber & Mohr (88% MLB) (Fig. 3). *Gracilaria chilensis* contained six *orf*s in three plasmids (Gch7220 [GI: 11465591], Gch3937 [GI: 11465608], and GC2 [GI: 18476]); however, those *orf*s did not group with the plastid-encoded homologs that were clustered (99% MLB) with the pseudogenized plastid gene of *G. tenuistipitata* (144 aa) and plasmid Gle4293 (GI: 11465614) *orf*1 of *G. lemaneiformis*.

Because the evolutionary trajectories of plasmid and plastid copies are very different (the former presumably functional and therefore subject to purifying selection, but the latter pseudogenized and under relaxed selective constraint), it is difficult to infer evolutionary relationships, since both rates and types of mutation (synonymous versus nonsynonymous) may be very different depending on the genetic background. We think it is likely that the plasmid *orfs* are ancestral because they contain complete *orfs* (405–485 aa), whereas plastids contain pseudogenized genes (up to 190 aa). On the other hand, plastid sequences occur in all the *Gracilaria* clades; the difference may be due to relaxed purifying selection on the shorter, non-functional (pseudogenized) plastid copies. Pp6427 *orf*3 homologs were found in the closely related genera *Grateloupia, Gracilaria,* and *Gracilariopsis* (multigene phylogeny using mitochondrial genes^43^), suggesting that an ancestral Pp6427 *orf*3 of *P. pulchra* was transferred into the ancestral plastid of these genera and the mitochondrial genome of *Palmaria palmata*. Some plasmid *orf*s were duplicated (e.g., Gch7220 *orf*s and Gch3937 *orf*1) and fragmented (*orf*1, *orf*6, and *orf*7) within a plasmid (Gch7220). Although the origin of the plasmid-derived sequences is unknown, they may have spread into red algal organelle genomes and subsequently undergone relaxed selective constraint.

Two other plasmid *orf*s of Pp6427, *orf*2 and *orf*4 showed exclusive homology to cyanobacteria and green plants species, respectively. Pp6427*orf*2 was homologous to a putative transcriptional regulator protein (GI: 495464247) from the cyanobacterium *Moorea producens* (*e*-value: 4*e*^−08^) and to other cyanobacterial genes. The red algal plasmid *orf*2 was likely transferred from cyanobacteria (Fig. 4; MLB 90% in basal clade). The combined region from Pp6427 *orf*4 (Supplementary Table S7, ML tree is not shown) and their flanking regions were homologous to a hypothetical protein (*orf*619) from the plastid genome of *Ettlia pseudoalveolaris* (T.R. Deason & H.C. Bold) J. Komárek (green alga; GI: 725650857; BLASTx result *e*-value: 5*e*^−12^) as well as *orf*436 of *Mankyua chejuensis* B.Y. Sun, M.H. Kim & C.H. Kim (fern; GI: 727397314; BLASTx result *e*-value: 7*e*^−05^). Therefore, *orf*s encoded in the plasmid Pp6427 originated from various sources, and some *orf*s were subsequently transferred to the red algal plastid and mitochondrial genomes. Both plasmid Pp6859 *orf*4-*orf*5 and Pp6427 *orf*2 were homologous to cyanobacterial *orfs*, including those from several common species, *Calothrix* sp. 336/3, *Moorea producens* 3L, and *Rivularia* sp. PCC7116. Thus, these two plasmids may have served as reservoirs for *orf*s from different sources that eventually were delivered to organelles.

### Bacterial and viral origins of red algal plasmid ORFs

Bacterial or viral sequences were detected by a BLASTp search of the NCBI (nr) database using 22 PD red algal plastid *orf* queries (Table S5). The homologous sequence of *Gracilariopsis lemaneiformis* plasmid GL3.5 *orf*2 in the *Grateloupia taiwanensis* plastid genome showed a close phylogenetic relationship with bacterial and viral sequences (Supplementary Fig. S8). This red algal clade was positioned within the bacterial clades (100% MLB), suggesting the bacterial origin of the GL3.5 *orf*2 homologs. It was, however, unclear whether this plasmid-related sequence was transferred from bacteria directly or by a virus-mediated process, because the clade showed a sister relationship to the viral clade but with weak statistical support (48% MLB).

The ML tree based on red algal plasmid-encoded replicase genes (i.e., *Pyropia tenera,* GI: 17980119, 254029132; *P. pulchra,* GI: 7108457, 7108459, 7108461) and homologous sequences from a BLASTp search suggest a viral origin of these plasmid *orf*s (see Supplementary Fig. S9). Five red algal plasmid *orf*s, mitochondrial *orf*98 from *Phytophthora sojae* (stramenopile; GI: 145932354), and nuclear-encoded genes from *Reticulomyxa filosa* (rhizaria; GI: 569382219, 569382219) grouped together with diverse circular virus DNA (52% MLB), as well as with obligate parasitic bacteria (i.e., onion yellow phytoplasma) (78% MLB). A BLASTp search (cutoff *e*-value ≤ 1.0*e*^−05^, see Table 1) recognized eight sequences from the nuclear genome of *Nicotiana tomentosiformis* (green plant; GI: 697190580, 1587991, 697190578, 697159806, 697175541, 697190576, 697140845, 697149473) that were distantly related to the red algal plasmid clade. The replicase gene from the *P. pulchra* plasmid (GI: 7108457) was reported as a geminivirus-related sequence because it share five conserved motifs and phylogenetic affinities^51^,^78^.

Virus-derived plasmid genes (i.e., GL3.5 *orf*2, three replicase genes in *P. pulchra* plasmids, and two replicase genes in *Py. tenera* plasmids) were detected in both eukaryotic nuclear and organellar genomes. These were different from non-viral-derived red algal plasmid homolog sequences that were found only in organelle genomes (Table 1). It is likely that virus-derived plasmid genes could be transferred to the eukaryotic nuclear genome more easily than could non-viral plasmid genes.

### Remnant DNA replication domain in plasmid-derived plastid genes

**Figure 5:**
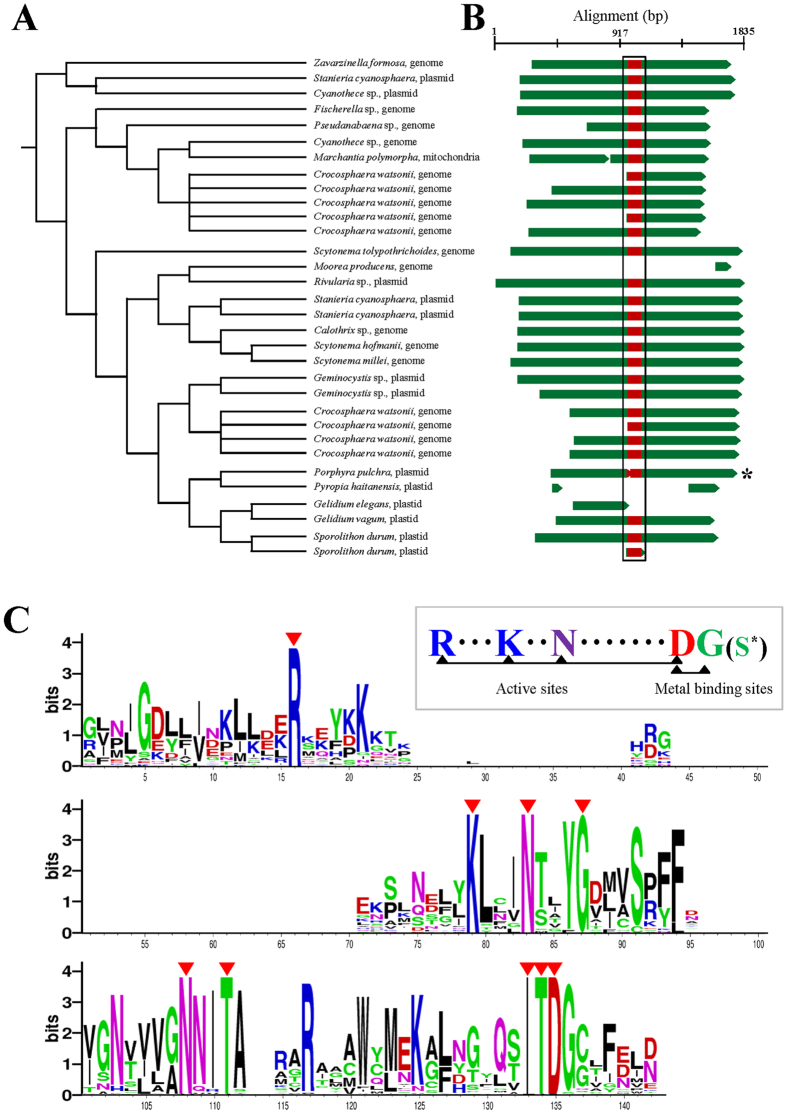
Domain search of *Porphyra pulchra* plasmid Pp6859 *orf*4-*orf*5 homolog. (**A**) Cladogram of the best maximum likelihood tree, Fig. 2. (**B**) Schematic of the DNA polymerase type-B family catalytic domain (POLBc) superfamily related regions (rectangular boundary) in the hypothetical homologous gene (green bars). Active and metal binding sites on the POLBc superfamily are shown below the alignment. In the domain of *Porphyra pulchra* (*), serine (S*) substitutes for glycine (G) in metal binding sites. (**C**) The composition of amino acids in alignment. Representative color of amino acid follows the Chemistry color scheme in WebLogo with probability-based size differences. The nine red arrowheads indicate conserved amino acids (100%) in the alignment. Blanks in the alignment indicate extremely low contribution by the hypothetical protein in *Fischerella* sp. (GI: 652333536) and in *Scytonema tolypothrichoides* (GI: 750920257).

**Figure 6:**
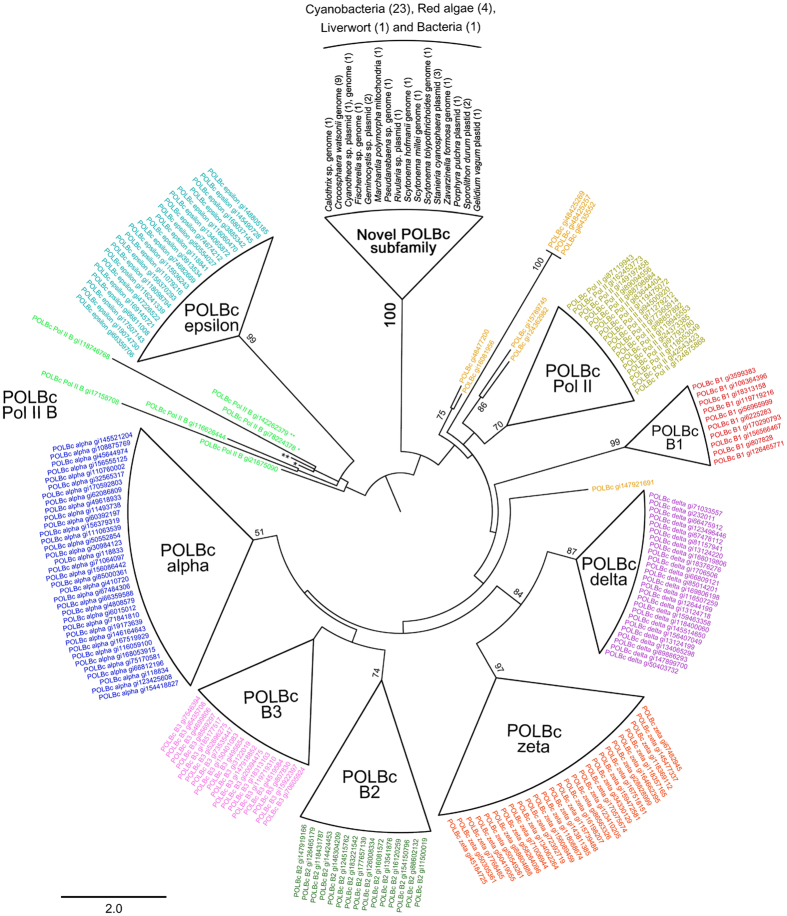
Maximum likelihood (ML) tree based on aligned amino acid sequences of DNA polymerase type-B family catalytic domain (POLBc) superfamily with 2,000 ML bootstrap replications. A public POLBc database was used from the conserved domain database (CDD) (Supplementary Table S8). Each cluster indicates a subfamily of POLBc superfamily. The novel POLBc subfamily clade comprises the POLBc-related partial domains of the hypothetical gene in this study. Eight public domain data are not identified to a specific subfamily in the POLBc superfamily (bright orange; GI: 16081956, 48477200, 15789745, 124362982, 48425269, 48425257, 6435552, 147921691). The DNA polymerase type-II B (Pol II B; bright green) subfamily is not monophyletic. Most subfamilies of POLBc are monophyletic; however, some inter-clade relationships differ slightly from the public POLBc superfamily database (CDD cl00145).

Plasmids are composed of three essential domains for replication, segregation and conjugation with additional accessory genes^76^,^77^. From the alignment of the Pp6859 *orf*4-*orf*5 homologs with the size range of 104 ~1,242 amino acid sequences, the functional domain was detected by a conserved domain database search^79^. One distinct domain is the DNA polymerase type-B family catalytic domain (POLBc) superfamily. Nine amino acid sequences were identical in this domain (aligned 142 aa), including highly conserved active sites (R-K-ND motif) and metal binding sites (DG motif) (see Fig. 5). The DNA polymerase type-B family consists of an editing active site and excision region for DNA replication (562 ~3,425 aa in size) that has been reported in a wide range of organisms, including Archaea, Bacteria, eukaryotes, bacteriophages and viruses^80^,^81^,^82^,^83^,^84^,^85^,^86^,^87^,^88^,^89^,^90^,^91^. Although the POLBc motif was generally conserved in nine major subfamilies^85^, we found differences in the catalytic domain of the Pp6859 *orf*4-*orf*5 homologs. These unique domains were represented in the ML tree that was reconstructed using homolog regions of the domain (aligned 222 aa) from the public POLBc superfamily database (Supplementary Table S8; Fig. 6). The ML tree showed that all POLBc domains in the Pp6859 *orf*4-*orf*5 homolog were grouped into a clade (100% MLB), but the clade did not belong to any other known POLBc subfamilies. This novel POLBc domain might contribute to the insertion of plasmid *orf*s into the red algal plastid genome.

## Conclusions

**Figure 7:**
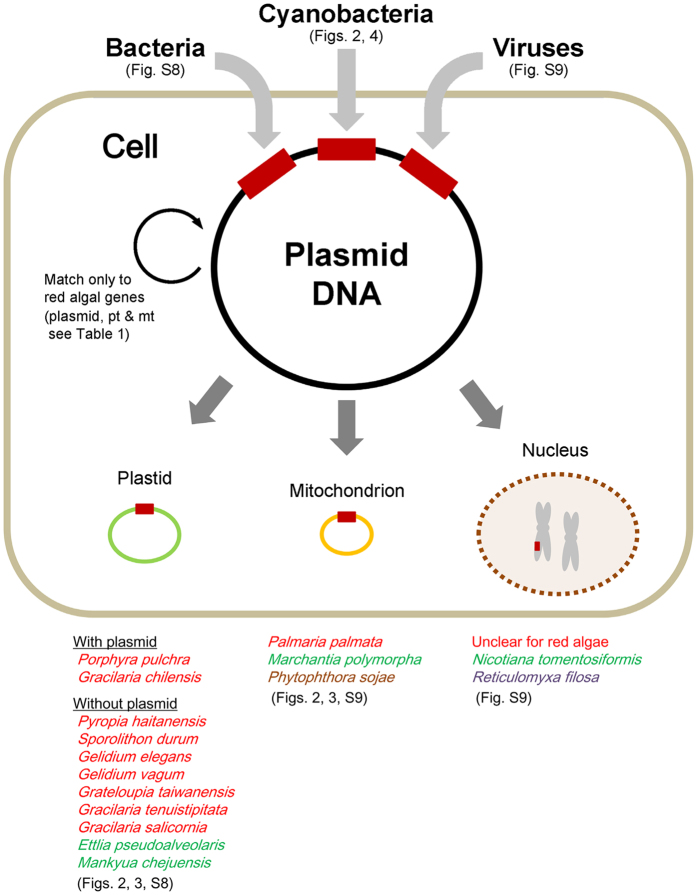
The spread of plasmid DNA in eukaryote genomes. The schematic cell includes the nucleus (dotted line circle), plastid (green circle), mitochondrion (orange circle) and plasmid (black circle) DNA. The plasmid-derived regions are indicated as red boxes in the genomes. The flow of plasmid DNA is indicated by the arrows. Plasmid-mediated HGT in plastid genomes are divided into two types: with plasmid and without plasmid (Figs 2, 3 and S8). Organisms with and without plasmid DNA are listed below as red algae (red taxa), green lineage (green taxa), stramenopiles (brown taxa), and rhizarians (violet taxa). Plastid genomes of *Porphyra pulchra* and *Gracilaria chilensis* include plasmid-derived homologs in both their plastid and plasmid genomes. The other red algae include plasmid-derived homologs only in the plastid genome. Mitochondrial HGT is found in red algae, the green lineage and stramenopiles (Figs 2, 3 and S9). Plasmid-mediated transfer to the nuclear genome is found only in *Nicotiana tomentosiformis* (plants) and *Reticulomyxa filosa* (rhizarian), with both regions related to viruses (Supplementary Fig. S9).

Plasmids have long been recognized as mobile elements but their origins in red algae remained unclear. Using a comprehensive database of 21 plastid genomes that included five novel red algal ptDNAs, we found evidence for the spread of plasmid DNA into plastid and mitochondrial genomes. There is currently insufficient nuclear genome data from species that contain plasmid-derived DNA to determine whether this compartment is also a major target for integration (Fig. 7). The distribution of plasmid-derived *orfs* showed a species-specific pattern, consistent with the evolution of a mobile genetic element. Because organelles are inherited maternally, foreign genetic DNA can be rapidly fixed in a population. Consistent with this idea, individual members of three lineages (i.e., *Porphyra pulchra, Sporolithon durum,* and *Gelidium elegans*) all showed plasmid DNA retention, although these *orfs* were absent or located in different genomic positions in closely related sister species (e.g., eight *Porphyra/Pyropia* species, *Sporolithon-Calliarthron*, two *Gelidium* species, see Fig. 1). It is known that the distribution of transposable elements can show variation within a single cyanobacterial species^72^,^73^,^92^. Therefore, plasmids may be regarded as analogous to transposable elements^76^,^77^,^93^,^94^,^95^,^96^, with mobility and loss contributing to variation in gain/loss among closely related genomes. For instance, Halary *et al.*^97^ demonstrated that plasmids are key vectors of genetic exchange between bacterial chromosomes on the basis of network analysis using sequences including phage, plasmid and environmental viral genomes.

It should be noted that we were originally interested in testing the idea whether plasmids may have facilitated EGT in algae and thereby played a key role in their genome evolution. Analysis of the available data, however, suggests that plasmids are better thought of as parasitic elements (e.g., group II introns in red algal ptDNA^98^) that spread plasmid-derived DNA regions. As “mobile gene cassettes”^75^,^76^,^77^,^78^ it nonetheless remains possible that these selfish elements can mediate gene transfer between foreign DNA and organelles. As the databases of available organelle and nuclear genome data increase, plasmid involvement in recent instances of EGT may become apparent.

In summary, one of the major challenges in the field of microbial eukaryote genome evolution is to understand how genes move across the tree of life. Species such as *Galdieria sulphuraria* encode at least 5% foreign genes, many of which are clearly of adaptive value^69^. The halotolerant green alga *Picochlorum* SE3 has acquired at least 24 genes of bacterial provenance, putatively to deal with abiotic stress^99^. Plasmids, viruses, symbionts, and pathogens likely all play a role in the HGT process in protists. Therefore, the search for “smoking guns” of recent transfer will continue to fascinate biologists who seek to show that highways of gene sharing^100^, common in prokaryotes, are drivers of evolution in eukaryotic microbes.

## Methods

### Sample preparation, genome sequencing, and assembly

Thalli of the red algal species *Gelidium elegans* Kützing, *G. vagum* Okamura, *Gracilaria chilensis* C.J. Bird, J.L. McLachlan, & E.C. de Oliveira, and *Sporolithon durum* (Foslie) R. Townsend & W. Woelkerling were collected from nature and immediately dried in silica-gel. Tissue samples of *Porphyra pulchra* were taken from herbarium specimens collected in 1970 and housed at the University Herbarium, University of California at Berkeley (UC). Detailed information about the samples is shown in Table S6 in the Supplementary Information. Genomic DNA was extracted using the DNeasy Plant Mini Kit (Qiagen, Hilden, Germany). Next-Generation Sequencing (NGS) was carried out using Ion Torrent PGM platform (Life Technologies, San Francisco, California, USA). The Ion Xpress Plus gDNA Fragment Library Kit (Life Technologies) was used for 200 bp-sized or 400 bp-sized sequencing library preparation. Genome sequencing was done with the Ion PGM Template OT2 200 or 400 Kit and Ion PGM Sequencing 200 or 400 Kit (Life Technologies, San Francisco, California, USA).

The raw NGS reads were assembled using the CLC Genomics Workbench 5.5.1 (CLC bio, Aarhus, Denmark) and the MIRA assembler that was incorporated in the Ion Server. Contigs of plastid genes were sorted by customized Python scripts with local BLAST searches. Sorted contigs were re-assembled to construct consensus plastid genomes. A draft plastid genome was confirmed by the read-mapping method using CLC Genomics Workbench 5.5.1. Gaps were filled by PCR to generate intact genomes.

### Gene annotation and plasmid-derived ORFs search

Putative ORFs in the five novel genomes were predicted using ORF Finder in Geneious 6.1.6^101^ and annotated based on BLASTx searches (*e*-value ≤ 1.0*e*^−05^) with codon table 11 (Bacterial, Archaeal and Plant Plastid Code). Ribosomal RNAs and transfer RNAs were predicted using the RNAmmer 1.2 Server^102^ and ARAGORN programs^103^. Group II intron and RNase P were searched using the program RNAweasel (http://megasun.bch.umontreal.ca/cgi-bin/RNAweasel/RNAweaselInterface.pl). Plasmid-derived sequences were searched by BLASTx (*e*-value ≤ 1.0*e*^−05^) using 56 proteins encoded in 14 red algal plasmids (Supplementary Table S9) derived from all available red algal ptDNAs. We also searched for plasmid-derived sequences in nuclear genome data. Here 56 plasmid-encoded genes were searched in the complete nuclear genomes of *Cyanidioschyzon merolae*^67^,^68^, *Galdieria sulphuraria* (Galdieri) Merola^69^, *Porphyridium purpureum* (Bory) K.M. Drew & R. Ross^70^, *Calliarthron tuberculosum* (Postels & Ruprecht) E.Y. Dawson^71^, *Chondrus crispus* Stackhouse^56^ and the 5 novel red algal draft genomes. Reported plasmid sequences from *Gracilaria chilensis* (GI: 11465591, 11465608 and 18476) and *Porphyra pulchra* (GI: 11466614, 11466608, 7108456, 7108458 and 7108460) were used as reference sequences for the read-mapping method for NGS data. To check consistency within individuals and populations, plasmid-derived sequences were determined from three individuals of *Gelidium elegans* from three different sites in Korea (SKKU18, SKKU22 and SKKU28), two individuals of *Porphyra pulchra* from Moss Beach, CA, USA (UC1454976 and UC1879714), and three individuals of *Sporolithon durum* from Army Bay, Whangaparaoa, NZ (SKKU_SD01, SKKU_SD02 and SKKU_SD03) using PCR with custom primer pairs (Supplementary Table S10).

### Phylogenetic analysis of red algal plasmid-derived genes in plastid genome

Plastid-coding genes from 21 taxa (16 reference genomes and our five new genomes) were extracted and sorted by customized Python scripts with local BLAST searches. To identify the independent loss of plastid genes, each gene set was manually analyzed. A selection of 193 plastid-coding genes (e.g., homologous genes present in at least 16 different taxa) and plasmid-derived sequences were aligned using MAFFT 7.110^104^. All aligned plastid genes were concatenated for multigene phylogenetic analysis. Based on the alignment, fragmented plasmid-derived *orf*s were combined (Supplementary Table S7). To reconstruct the phylogenetic tree, an evolutionary model was selected using Modeltest implemented in MEGA 6.0^105^. Maximum likelihood (ML) tree search and ML bootstrap analysis were done using RaxML 8.0.0 with 2000 replications^106^ with the PROT + GAMMA + LG4MF model of evolution.

### Domain prediction and phylogenetic analysis

Protein domain prediction was done using the conserved domain database CDD^79^. Predicted domain sequences were aligned and represented using Weblogo^107^. Aligned sequences of DNA polymerase type-B family catalytic domain (POLBc) subfamilies (POLBc_B1, POLBc_B2, POLBc_alpha, POLBc_delta, POLBc_zeta, POLBc_epsilon, POLBc_B3, POLBc_Pol_II, POLBc_Pol_II_B and unspecified POLBc domain (Supplementary Table S8) were used to find the inter-subfamily relationship based on the RAxML phylogeny.

## Additional Information

**How to cite this article**: Lee, J.M. *et al.* Reconstructing the complex evolutionary history of mobile plasmids in red algal genomes. *Sci. Rep.* **6**, 23744; doi: 10.1038/srep23744 (2016).

## Supplementary Material

Supplementary Information

Supplementary Information 2

## Figures and Tables

**Table 1:**
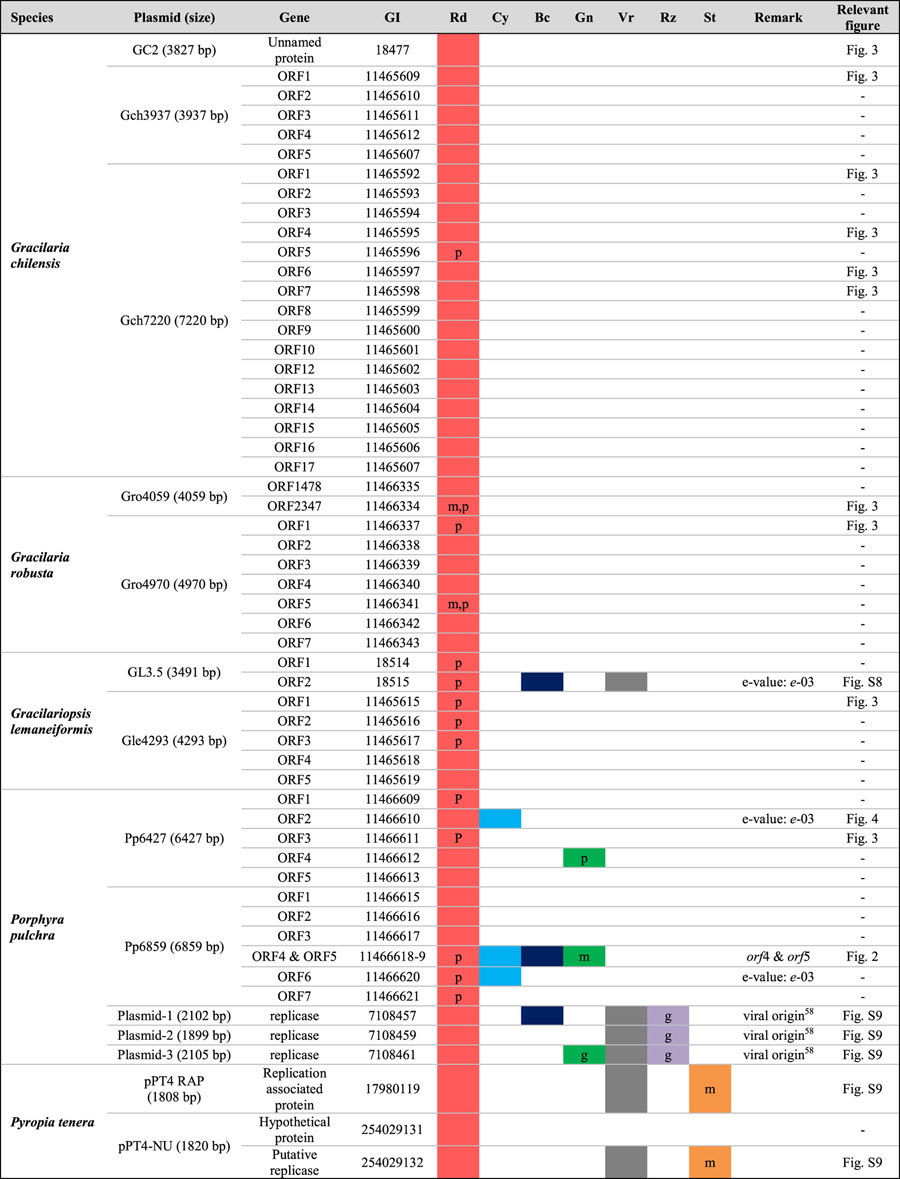
Distribution of red algal plasmids and their homologous sequences (BLASTp results, cut-off = *e*^−05^).

Rd = red algae, Cy = Cyanobacteria, Bc = Bacteria (excluding Cyanobacteria), Gn = green plant lineage (Viridiplantae), Vr = Virus, Rz = Rhizaria, St = Stramenopile. Letters beside the filled circles indicate origins: *m* for the mitochondrial homolog, *p* for the plastid homolog and *g* for the nuclear genome homolog. When the letters m, p, or g are absent, the origin of the gene is unknown.
