# Supplementary material for: Reconstructing the complex evolutionary history of mobile plasmids in red algal genomes
Source: Sci Rep. 2016 Mar 31;6:23744. doi: 10.1038/srep23744 (PMC4814812; doi:10.1038/srep23744)
Supplement: Supplementary Information [file srep23744-s1.pdf]

Supplementary information

Reconstructing the complex evolutionary history of mobile plasmids in red algal genomes

JunMo Lee[1], Kyeong Mi Kim[2], Eun Chan Yang[3], Kathy A. Miller[4], Sung Min Boo[5], Debashish Bhattacharya[6], & Hwan Su Yoon[1,*]

List of Tables

Table S1. Information about the NGS data for five newly sequenced red algal plastid genomes.

Table S2. Gene compositions of red algal plastid genomes.

Table S3. List of 193 plastid protein-coding genes used in the ML tree search.

Table S4. Newly annotated genes from previously published red algal plastid genomes. These genes have been overlooked, underestimated or mis-annotated in previous studies.

Table S5. Results of BLASTx search using plasmid-derived regions in plastid genomes. Cutoff of *e*-value is $1.0e^{-05}$ and two cases of relaxed *e*-value ($2.0e^{-01}$ and $3.0e^{-03}$) are adapted to Gle4293 *orf*4 of *Sporolithon* and Gro4059 *orf*2347 of *Grateloupia*. Abbreviations: HyP indicates hypothetical protein as in *orf* for the open reading frame, PG for the pseudogenized gene. In the plastid genome region of *Sporolithon durum,* between *arg*B and *fts*H gene, 4.2 kb (non-coding) includes two plasmid-derived pseudogenized genes. In the plastid genome of *S. durum*, the region between rRNAs and *ycf*27 pm_*orf*86 shows uncertain mixed plasmid signals from BLASTx search results. The plastid genome of *Gracilariopsis tenuistipitata* var. *liui* also shows uncertain plasmid signals between *leu*D and *psb*D gene.

Table S6. Samples used in this study.

Table S7. Combined and pseudogenized cases of plasmid-derived regions. Several partial regions of plasmid-derived regions were combined into each single or pseudogenized gene based on their alignment as complementary parts. Detailed information about these sequences is shown below the table. In the genome of *Moorea producens*, hypothetical protein (GI: 332351884) and both flanking regions were combined and these bacterial sequences were translated using the Bacterial, Archaeal and Plant Plastid Code (codon table 11). In the mitochondrial genome of *Marchantia polymorpha*, *orf*196 (GI: 11467138) and *orf*207 (GI: 11467137) were combined with their flanking non-coding regions respectively, and their two regions were also combined as a single pseudo-gene (89 bp interval between two regions). For phylogenetic analysis, these plant mitochondrial sequences have been translated to amino acids using a standard code (codon table 1). In red algae, each part of pseudo-gene regions was translated using the codon table 11 and combined serially.

Table S8. Contents of subfamily groups in DNA polymerase type-B family catalytic domain (POLBc) superfamily from conserved domain database (CDD).

Table S9. List of published red algal plasmids. 13 species out of a total 36 red algal species surveyed contain plasmids (marked as "present [O]"). Among 13 species, 14 plasmid sequences were reported from only 5 species (*Porphyra pulchra*, *Pyropia tenera*, *Gracilaria chilensis*, *Gracilaria robusta* and *Gracilariopsis lemaneiformis*) in the NCBI database. 14 plasmid sequences encoded 56 open-reading frames (*orf*).

Table S10. Primer information for population analysis. Different individuals of *Gelidium elegans*, *Porphyra pulchra* and *Sporolithon durum* were used to check the presence or absence of the plasmid-derived region. The plasmid-derived regions of *G. elegans* (between *ycf*27 and *psb*D gene), *P. pulchra* (between *orf*121 and *apc*F gene) and *S. durum* (between pseudogenized *syf*B and *rps*1 gene) were confirmed by PCR.

List of Figures

Fig. S1. Plastid genome map of *Porphyra pulchra*. Functional gene groups are color-coded. Genes drawn inside of the circle indicate the transcriptionally clockwise strand, and genes on outside indicate the counter-clockwise strand.

Fig. S2. Plastid genome map of *Sporolithon durugm*. Functional gene groups are color-coded. Genes drawn inside of the circle indicate the transcriptionally clockwise strand, and genes on outside indicate the counter-clockwise strand.

Fig. S3. Plastid genome map of *Gelidium elegans*. Functional gene groups are color-coded. Genes drawn inside of the circle indicate the transcriptionally clockwise strand, and genes on outside indicate the counter-clockwise strand.

Fig. S4. Plastid genome map of *Gelidium vagum*. Functional gene groups are color-coded. Genes drawn inside of the circle indicate the transcriptionally clockwise strand, and genes on outside indicate the counter-clockwise strand.

Fig. S5. Plastid genome map of *Gracilaria chilensis*. Functional gene groups are color-coded. Genes drawn inside of the circle indicate the transcriptionally clockwise strand, and genes on outside indicate the counter-clockwise strand.

Fig. S6. Maximum likelihood tree based on aligned amino acid sequences of concatenated 193 protein encoding genes from 16 published and 5 new (underlined) plastid genomes (2000 replications, bootstrap support > 50%).

Fig. S7. Synteny of all plasmid-derived regions in red algae. Colored boxes indicate each homolog region in red algal plasmid and plastid genomes. *Porphyra pulchra* plasmid Pp6859 *orf*4 and *orf*5 homolog regions are also presented with two cyanobacterial species.

Fig. S8. Maximum likelihood tree based on aligned amino acid sequences of *Gracilariopsis lemaneiformis* plasmid GL3.5 *orf*2 related genes and its homologs from BLASTp search (cutoff *e*-value $1.0e^{-03}$) (2000 replications, bootstrap support > 50%). Taxon information and their sequence localities are color-coded as follows: bacteria (black), red algae (red), virus (violet), plastid (green) and plasmid (brown).

Fig. S9. Maximum likelihood tree based on aligned amino acid sequences of red algal replicase genes from *Pyropia tenera* (GI: 254029132, 17980119) plasmids, *Porphyra pulchra* plasmid (GI: 7108461, 7108459, 7108457) and their homologs from BLASTp search (cutoff *e*-value $1.0e^{-05}$) (2000 replications, bootstrap support > 50%). Taxon information and their sequence localities were indicated with color-code as follows: parasitic bacteria (blue), plant (green), red algae (red), rhizaria (cyan), stramenopile (brown), plasmid (violet), mitochondria (orange) and nucleus (black).

Table S1. Information about the NGS data for five newly sequenced red algal plastid genomes.

No.	Taxa	Total bases of NGS sequencing data (Mbp)	Size of complete plastid genome (bp)	Total bases of mapped plastid genome (bp)	Average coverage of plastid genome (X)
1	*Gelidium elegans*	1,529 Mbp	174,749 bp	44,027,950 bp	228X
2	*Gelidium vagum*	990 Mbp	179,852 bp	66,802,278 bp	339X
3	*Gracilaria chilensis*	506 Mbp	185,637 bp	87,006,562 bp	445X
4	*Porphyra pulchra*	263 Mbp	194,175 bp	12,007,987 bp	52X
5	*Sporolithon durum*	3,190 Mbp	191,464 bp	32,008,868 bp	159X

Table S2. Gene compositions of red algal plastid genomes.

Species	Reference	GC (%)	Coding genes	tRNAs	rRNAs	Introns
**Cyandiophyceae**						
Cyanidioschyzon merolae (NC_004799)	Ohta et al. 2003	37.6	207	31	3	0
Cyanidium caldarium (NC_001840)	Glöckner et al. 2000	32.7	199 (2*)	30	3	0
Galdieria sulphuraria (NC_024665)	Jain et al. 2014	28.5	182	39	6	0
**Porphyridiophyceae**						
Porphyridium purpureum (NC_023133)	Tajima et al. 2014	30.3	224	29	6	43
**Bangiophyceae**						
Porphyra pulchra	**This study**	33.3	207	37	6	0
Porphyra purpurea (NC_000925)	Reith & Munholland 1995	33.0	209	37	6	0
Porphyra umbilicalis (JQ408795)	Smith et al. 2012	32.9	208 (1*)	37	6	0
Pyropia fucicola (KJ776837)	Hughey et al. 2014	32.7	207 (4*)	35	3	0
Pyropia haitanensis (NC_021189)	Wang et al. 2013	33.0	213 (2*)	37	6	0
Pyropia kanakaensis (KJ776836)	Hughey et al. 2014	32.8	210 (4*)	35	3	0
Pyropia perforata (NC_024050)	Hughey et al. 2014	32.9	211 (2*)	35	3	0
Pyropia yezoensis (NC_007932)	Wang et al. 2013	33.1	213	37	6	0
**Florideophyceae**						
Calliarthron tuberculosum (NC_021075)	Janouškovec et al. 2013	29.2	201	31	3	2
Sporolithon durum	**This study**	29.3	202	30	3	2
Chondrus crispus (NC_020795)	Janouškovec et al. 2013	28.7	204	30	3	1
Gelidium elegans	**This study**	30.2	202	30	3	1
Gelidium vagum	**This study**	29.9	201	30	3	1
Gracilaria chilensis	**This study**	29.3	203	30	3	1
Gracilaria salicornia (NC_023785)	Campbell et al. 2014	28.8	203 (1*)	30 (1*)	3	1 (1*)
Gracilaria tenuistipitata var. *liui* (NC_006137)	Hagopian et al. 2004	29.2	205 (2*)	30 (1*)	3	1 (1*)
Grateloupia taiwanensis (NC_021618)	DePriest et al. 2013	30.6	234 (1*)	30 (1*)	3	1 (1*)

*: including un-annotated genes in previous reports

Table S3. List of 193 plastid protein-coding genes used in the ML tree search.

See the file “Table_S3_S4_S7_supplementary_tables.xlsx” (sheet Table S3).

Table S4. Newly annotated genes from previously published red algal plastid genomes. These genes have been overlooked, underestimated or mis-annotated in previous studies. Detailed information is shown in remarks.

See the file “Table_S3_S4_S7_supplementary_tables.xlsx” (sheet Table S4).

Table S5. Results of BLASTx search using plasmid-derived regions in plastid genomes. Cutoff of *e*-value is $1.0e^{-05}$ and two cases of relaxed *e*-value ($2.0e^{-01}$ and $3.0e^{-03}$) are adapted to Gle4293 *orf*4 of *Sporolithon* and Gro4059 *orf*2347 of *Grateloupia*. Abbreviations: HyP indicates hypothetical protein same as in *orf* for the open reading frame, PG for the pseudogenized gene. The plastid genome region of *Sporolithon durum* between *arg*B and *fts*H gene is 4.2 kb non-coding part that includes two plasmid-derived pseudogenized genes. Between rRNAs and *ycf*27 in plastid genome of *S. durum*, pm_*orf*86 shows uncertain mixed plasmid signals from BLASTx search results. The plastid genome of *Gracilariopsis tenuistipitata* var. *liui* also shows uncertain plasmid signals between *leu*D and *psb*D gene.

Species	Locality	Region	Feature	Size	Matched results	E-value	Identities	GI	Remarks
Porphyra pulchra	*orf*121-*apc*F	-	PG	585 bp	Pp6427 *orf*3	1.E-39	45%	11466611	
Pyropia haitanensis	*pet*G-*rps*14	-	PG	414 bp	Pp6859 *orf*7	3.E-44	63%	11466621	
	*pet*G-*rps*14	-	PG	291 bp	Pp6859 *orf* 6	3.E-22	48%	11466620	
	*pet*G-*rps*14	pm_*orf*72	*orf*	216 bp	Pp6859 *orf* 5	9.E-12	55%	11466341	un-annotated gene previously
	*ycf*12-*ftr*B	-	PG	399 bp	Pp6859 *orf* 4	4.E-36	51%	11466618	
	*ycf*12-*ftr*B	pm_*orf*93	*orf*	279 bp	Pp6427 *orf* 1	1.E-12	59%	11466609	un-annotated gene previously
Sporolithon durum	PG(*syf*B)-*rps*1	pm_*orf*185	*orf*	555 bp	Gle4293 *orf* 4	2.E-01	26%	11465618	relaxed e-value, Pp6859 *orf*1 region mixed
	PG(*syf*B)-*rps*1	pm_*orf*73	*orf*	219 bp	Pp6859 *orf* 6	4.E-06	37%	11466620	
	PG(*syf*B)-*rps*1	pm_*orf*819	*orf*	2457 bp	*Calothrix* sp. HyP	1.E-146	36%	668693613	Pp6859 *orf*4 and *orf*5 homolog
	*arg*B-*fts*H	-	PG	312 bp	*Calothrix* sp. HyP	8.E-09	78%	668693613	copy region of pm_*orf*819
	*arg*B-*fts*H	-	PG	381 bp	Gch7220 *orf* 5	5.E-14	36%	11465596	
Gelidium elegans	*ycf*27-*psb*D	-	PG	831 bp	Pp6859 *orf* 5	2.E-38	36%	11466341	
Gelidium vagum	*rps*6-rRNAs	-	PG	2091 bp	*Calothrix sp.* HyP	2.E-107	34%	66863613	Pp6859 *orf*4 and *orf*5 homolog
	*rps*6-rRNAs	-	PG	555 bp	Gro4970 *orf* 5	2.E-12	25%	11466341	
Grateloupia taiwanensis	*ycf*27-*psb*D	-	PG	306 bp	GL3.5 *orf* 1	1.E-05	35%	18514	overlapped to unique *orf*31 & *orf*32
	rRNAs-*ycf*27	-	PG	183 bp	GL3.5 *orf* 2	3.E-12	74%	18515	overlapped to unique *orf*06
	*ycf*27-*psb*D	-	PG	303 bp	Gro4059 *orf* 2347	3.E-03	27%	11466334	relaxed e-value, overlapped to unique *orf*32
Gracilaria salicornia	*ycf*27-*psb*D	*orf*139	*orf*+PG	495 bp	Gro4970 *orf* 1	3.E-24	50%	11466337	
Gracilaria tenuistipitata var. *liui*	rRNAs-*ycf*27	-	PG	441 bp	Gle4293 *orf* 1	5.E-14	64%	11465615	
Gracilaria chilensis	*leu*D-*psb*D	-	PG	606 bp	Gle4293 *orf* 1	7.E-37	61%	11465615	
	*leu*D-*psb*D	-	PG	300 bp	Gle4293 *orf* 2	6.E-08	36%	11465616	
	*leu*D-*psb*D	-	PG	408 bp	Gle4293 *orf* 3	1.E-23	52%	11465617	

Table S6. Sampling information used in this study.

No.	Taxa	Strain No.	Date	Locality
1	*Gelidium elegans*	SKKU18	Oct. 27, 2012	Anin, Gang-reung, Korea
		SKKU22	Oct. 27, 2012	Gamchusa, Donghae-si, Korea
		SKKU28	Oct. 22, 2012	Wando, Jungdo-ri, Korea
2	*Gelidium vagum*	-	Jul. 23, 2012	Manripo, Tae-an, Korea
3	*Gracilaria chilensis*	-	Feb. 1, 2003	Ancud, Chile
4	*Porphyra pulchra*	UC1879714	Jun. 20, 1970	Moss Beach, California, USA
		UC1454976	Jun. 20, 1970	Moss Beach, California, USA
5	*Sporolithon durum*	SKKU_SD01	Nov. 6, 2012	Army Bay, Whangaparaoa, New Zealand
		SKKU_SD02	Aug. 28, 2014	Army Bay, Whangaparaoa, New Zealand
		SKKU_SD03	Aug. 28, 2014	Army Bay, Whangaparaoa, New Zealand

Table S7. Information about the combination and separation of plasmid-derived regions. Several partial *orf* regions were combined into one single *orf*, while an *orf* was separated into two genes based on the complementary alignment. In the genome of *Moorea producens*, hypothetical protein (GI: 332351884) and its flanking regions (both of sides) were combined and these bacterial sequences were translated using the Bacterial, Archaeal and Plant Plastid Code (codon table 11). In the mitochondrial genome of *Marchantia polymorpha*, orf196 (GI: 11467138) and orf207 (GI: 11467137) were combined with their flanking non-coding regions, respectively, and their two regions were also combined as a single orf (89 bp interval between two regions). For phylogenetic analysis, these plant mitochondrial sequences have been translated to amino acids using a standard code (codon table 1). In red algae, each part of sequences were translated. All these red algal sequences were translated using the codon table 11.

See the file "Table_S3_S4_S7_supplementary_tables.xlsx" (sheet Table S7).

Table S8. Contents of subfamily groups in DNA polymerase type-B family catalytic domain (POLBc) superfamily from conserved domain database (CDD).

Conserved domain database	Accession
POLBc superfamily	cl10023
DNA polymerase type-B family catalytic domain (POLBc)	cd00145
DNA polymerase type-B B1 subfamily catalytic domain (POLBc B1)	cd05530
DNA polymerase type-B B2 subfamily catalytic domain (POLBc B2)	cd05531
DNA polymerase type-B alpha subfamily catalytic domain (POLBc alpha)	cd05532
DNA polymerase type-B delta subfamily catalytic domain (POLBc delta)	cd05533
DNA polymerase type-B zeta subfamily catalytic domain (POLBc zeta)	cd05534
DNA polymerase type-B epsilon subfamily catalytic domain (POLBc alpha)	cd05535
DNA polymerase type-B B3 subfamily catalytic domain (POLBc B3)	cd05536
DNA polymerase type-II subfamily catalytic domain (POLBc Pol II)	cd05537
DNA polymerase type-II B subfamily catalytic domain (POLBc Pol II B)	cd05538

Table S9. List of published red algal plasmids. 13 species out of a total 36 red algal species surveyed contain plasmids (marked as "present [O]"). Among 13 species, 14 plasmid sequences were reported from only 5 species (*Porphyra pulchra*, *Pyropia tenera*, *Gracilaria chilensis*, *Gracilaria robusta* and *Gracilariopsis lemaneiformis*) in NCBI database. 14 plasmid sequences encoded 56 open-reading frames (*orf*).

Order	Species	Present (O), Absent (X)	No. of plasmids	Reported plasmid sequences	NCBI GI (retained proteins)	References
Erythropeltidales	*Smithora naiadum*	O	3	X		Goff and Coleman 1990
Glaucosphaerales	*Glaucosphaera vaculolata*	X				Goff and Coleman 1990
Bangiales	*Porphyra linearis*	X				Villemur 1990a
	Porphyra pulchra	O	5	5	11466614 (7), 11466608 (5), 7108460 (1), 7108458 (1), 7108456 (1)	Moon and Goff 1996, 1997
	Pyropia perforata	X				Goff and Coleman 1990
	Pyropia tenera	O	?	2	17980118 (1), 254029130 (2)	Choi et al. 2001
	Wildemania miniata	O	3	X		Villemur 1990a
Ceramiales	*Asterocolax gardneri*	X				Goff and Coleman 1990
	Campylaephora californica	X				
	Choreocolax polysiphoniae	X				
	Harveyella mirabilis	X				
	Janczewski gardneri	X				
	Micorcladia coulteri	X				
	Odonthalia floccosa	X				
	Odonthalia washigtoniensis	X				
	Osmundea spectabilis	O	?	X		
	Polyneura latissima	X				
	Polysiphonia elongata	X				
	Vertebrata lanosa	X				
Gigartinales	*Gardneriella tubifera*	X				Goff and Coleman 1990
	Gymnogongrus sp.	O	2	X		Villemur 1990a
	Sarcodiotheca gaudichaudii	X				Goff and Coleman 1990
Gracilariales	*Gracilaria chilensis* (1)	O	4	1	18476 (1)	Villemur 1990a
	Gracilaria chilensis (2)		?	2	11465608 (5), 11465591 (16)	Goff and Moon (unpublished)
	Gracilaria pacifica	O	4	X		Goff and Coleman 1990
	Gracilaria robusta (1)	O	?	2	11466336 (7), 11466333 (2)	Goff and Moon (unpublished)
	Gracilaria robusta (2)		1	X		Goff and Coleman 1990
	Gracilaria textorii	O	2	X		
	Gracilaria tikvahiae	O	3	X		Villemur 1990a
	Gracilariophila oryzoides	O	2	X		Goff and Coleman 1990
	Gracilariopsis langissima	X				Villemur 1990a
	Gracilariopsis lemaneiformis	O	3	2	18513 (2), 11465614 (5)	Goff and Coleman 1990
	Gracilariopsis sp.	X				Villemur 1990a
Halymeniales	*Prionitis lanceolata*	X				Goff and Coleman 1990
Plocamiales	*Plocamiocolax pulvinata*	X				Goff and Coleman 1990
	Plocamium cartilagineum	X				
Rhodymeniales	*Lomentaria divaricata*	X				Goff and Coleman 1990

Table S10. Primer information for population analysis. Different individuals of *Gelidium elegans*, *Porphyra pulchra* and *Sporolithon durum* were used to check the presence or absence of plasmid-derived region. The plasmid-derived regions of *G. elegans* (between *ycf*27 and *psb*D gene), *P. pulchra* (between *orf*121 and *apc*F gene) and *S. durum* (between pseudogenized *syf*B and *rps*1 gene) were confirmed by PCR.

Speices	Strain	Primer set
Gelidium elegans	SKKU18<br>SKKU22<br>SKKU28	[Forward 1]<br>Gele_F1_1: 5'-ATTCAGTCTTCTAGAACTACTAGTAA-3'<br>Gele_F1_2: 5'-AAAGACATGTTGATACAAGAGTTGTC-3'<br><br>[Reverse 1]<br>Gele_R1_1: 5'-CTTGTCCAATTGCTATGGTCATATTA-3'<br>Gele_R1_2: 5'-AACCTTCTAGATAAGAACTAGCTAA-3'
Porphyra pulchra	UC1879714<br>UC1454976	[Forward 1]<br>Pp_F1_1: 5'-TCTAGATTATAGATAGATTTAGTGCTC-3'<br>Pp_F1_2: 5'-AAGACTAGTATATTTGTTAGTGCATCA-3'<br><br>[Reverse 1]<br>Pp_R1_1: 5'-AACTTTGAAGCTTTATTAGATGCAAGA-3'<br>Pp_R1_2: 5'-GCTTAGGATATTAGAATTCACAGATAA-3'
Sporolithon durum	SKKU_SD01<br>SKKU_SD02<br>SKKU_SD03	[Forward 1]<br>Spo_F1: 5’-CTGACACTGAGACAGATATAGAAAATTA-3'<br>[Reverse 1]<br>Spo_R1: 5’-AATTATTCATCGATCTTTCATTAGACAC-3'<br><br>[Forward 2]<br>Spo_F2: 5’-GTGCTAGTGTATTTAAGCTATATGAAG-3'<br>[Reverse 2]<br>Spo_R2: 5’-TTATTAAATAGAAGTTTAGCTCCATCT-3'

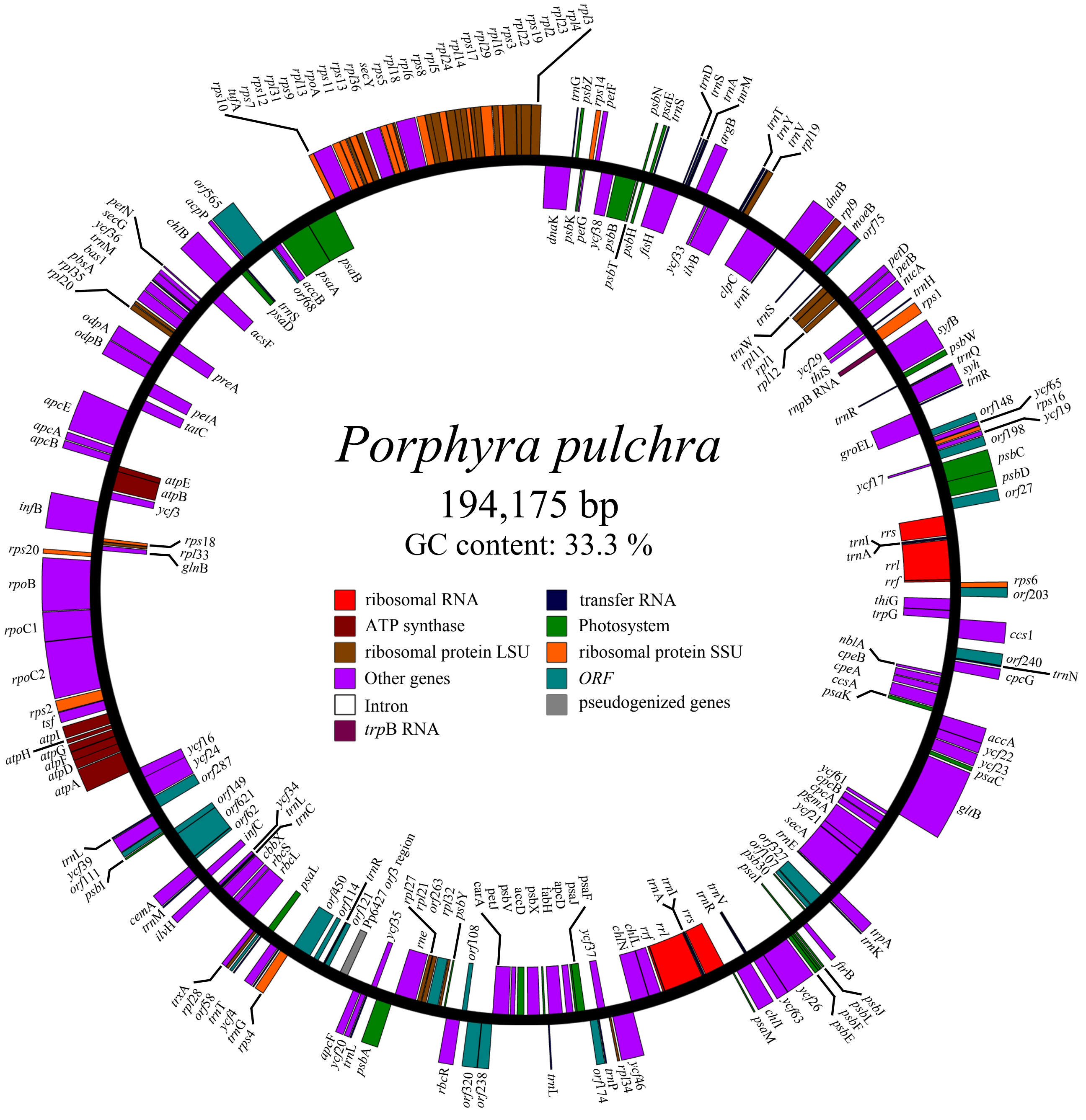

Fig. S1. Plastid genome map of *Porphyra pulchra*. Functional gene groups are color-coded. Genes drawn inside of the circle indicate the transcriptionally clockwise strand, and genes on outside indicate the counter-clockwise strand.

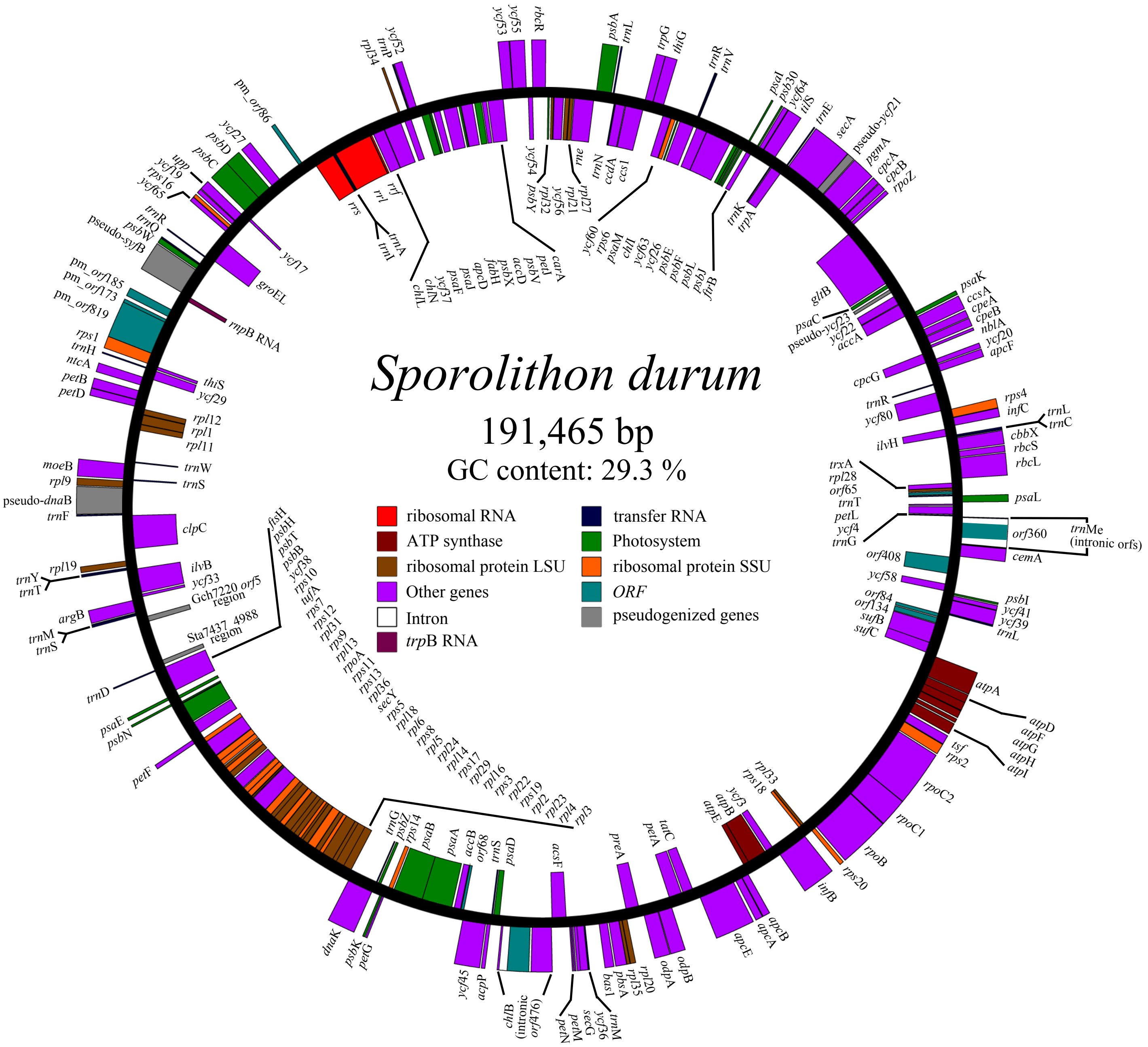

Fig. S2. Plastid genome map of *Sporolithon durum*. Functional gene groups are color-coded. Genes drawn inside of the circle indicate the transcriptionally clockwise strand, and genes on outside indicate the counter-clockwise strand.

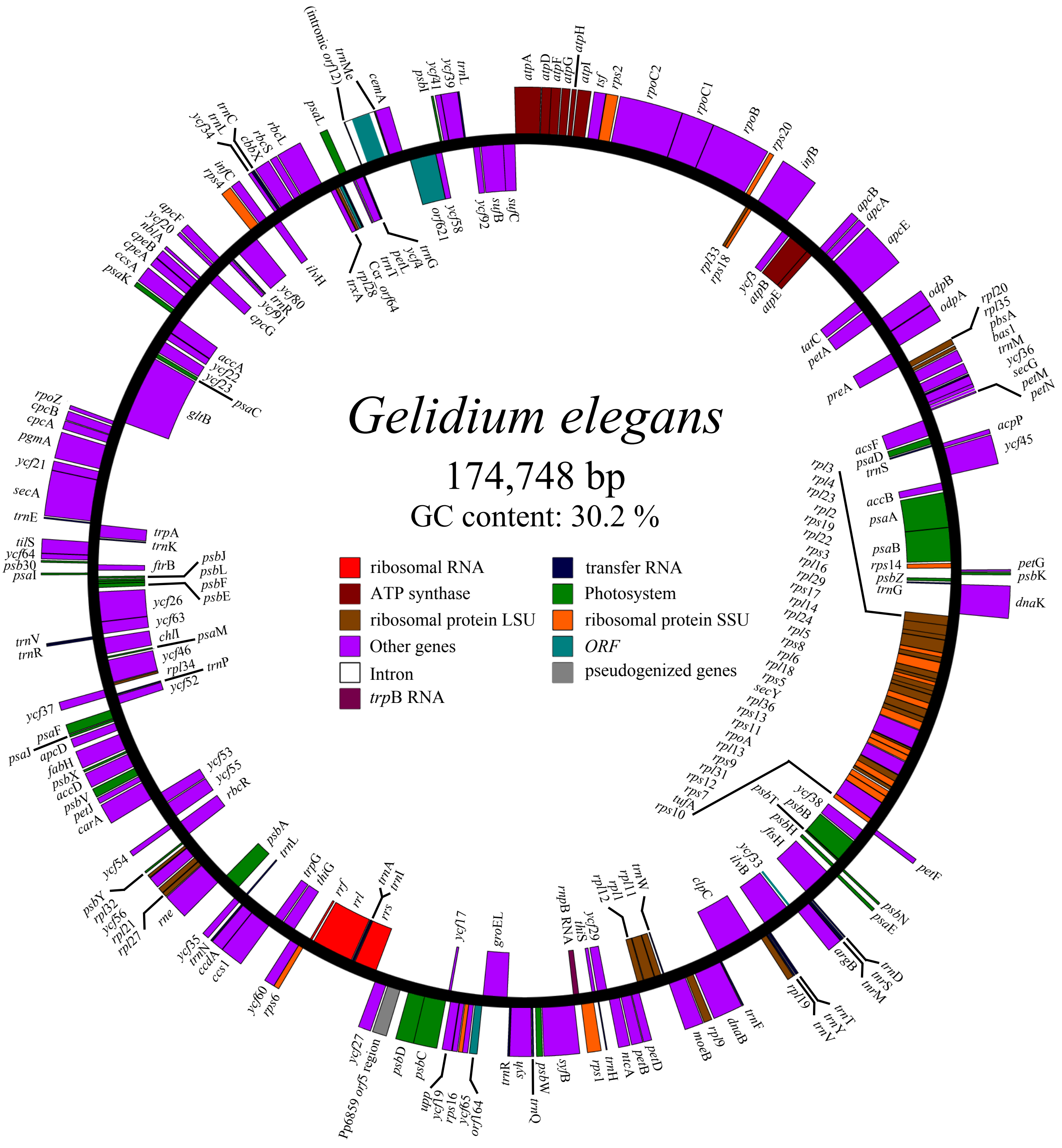

Fig. S3. Plastid genome map of *Gelidium elegans*. Functional gene groups are color-coded. Genes drawn inside of the circle indicate the transcriptionally clockwise strand, and genes on outside indicate the counter-clockwise strand.

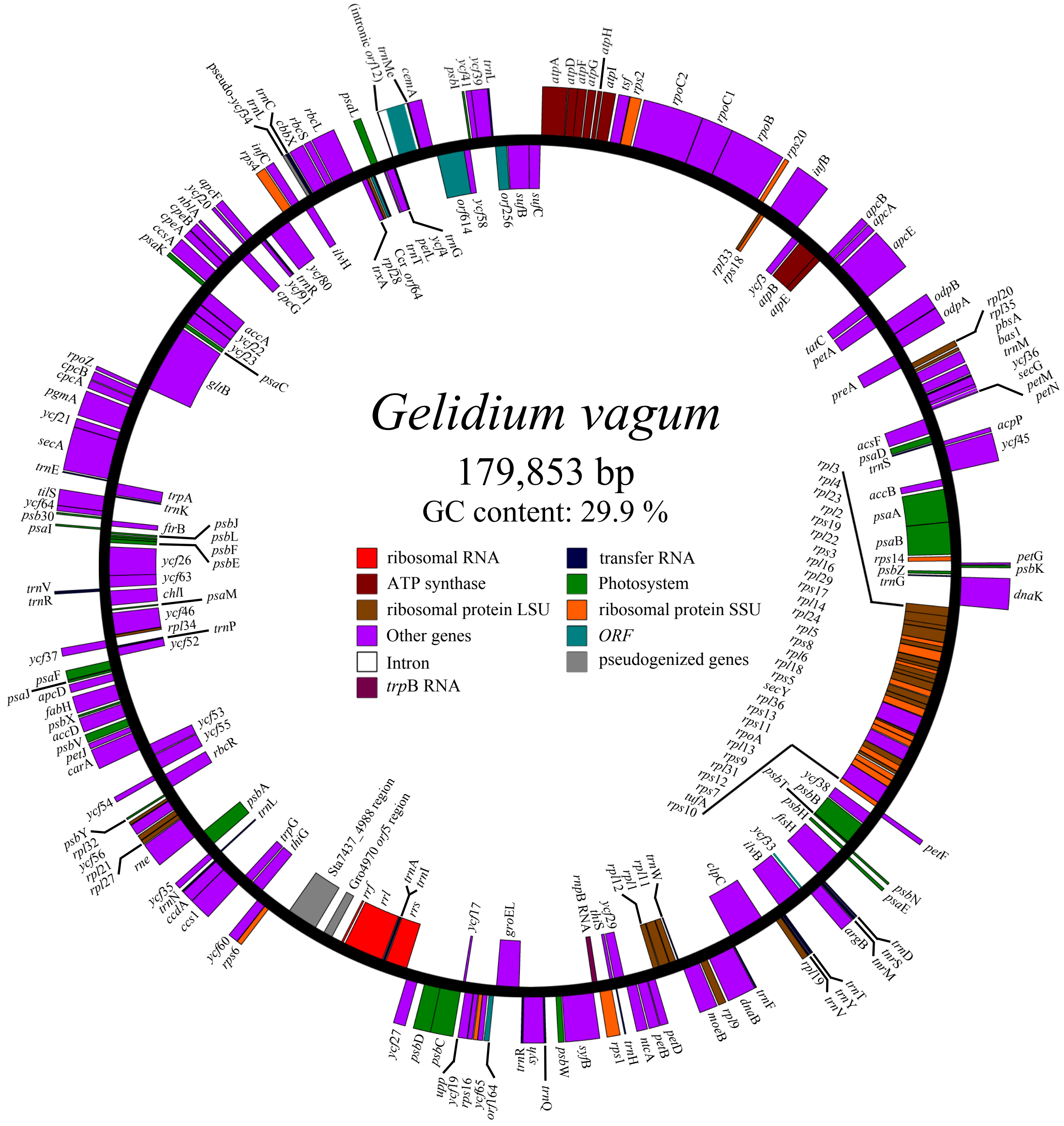

Fig. S4. Plastid genome map of *Gelidium vagum*. Functional gene groups are color-coded. Genes drawn inside of the circle indicate the transcriptionally clockwise strand, and genes on outside indicate the counter-clockwise strand.

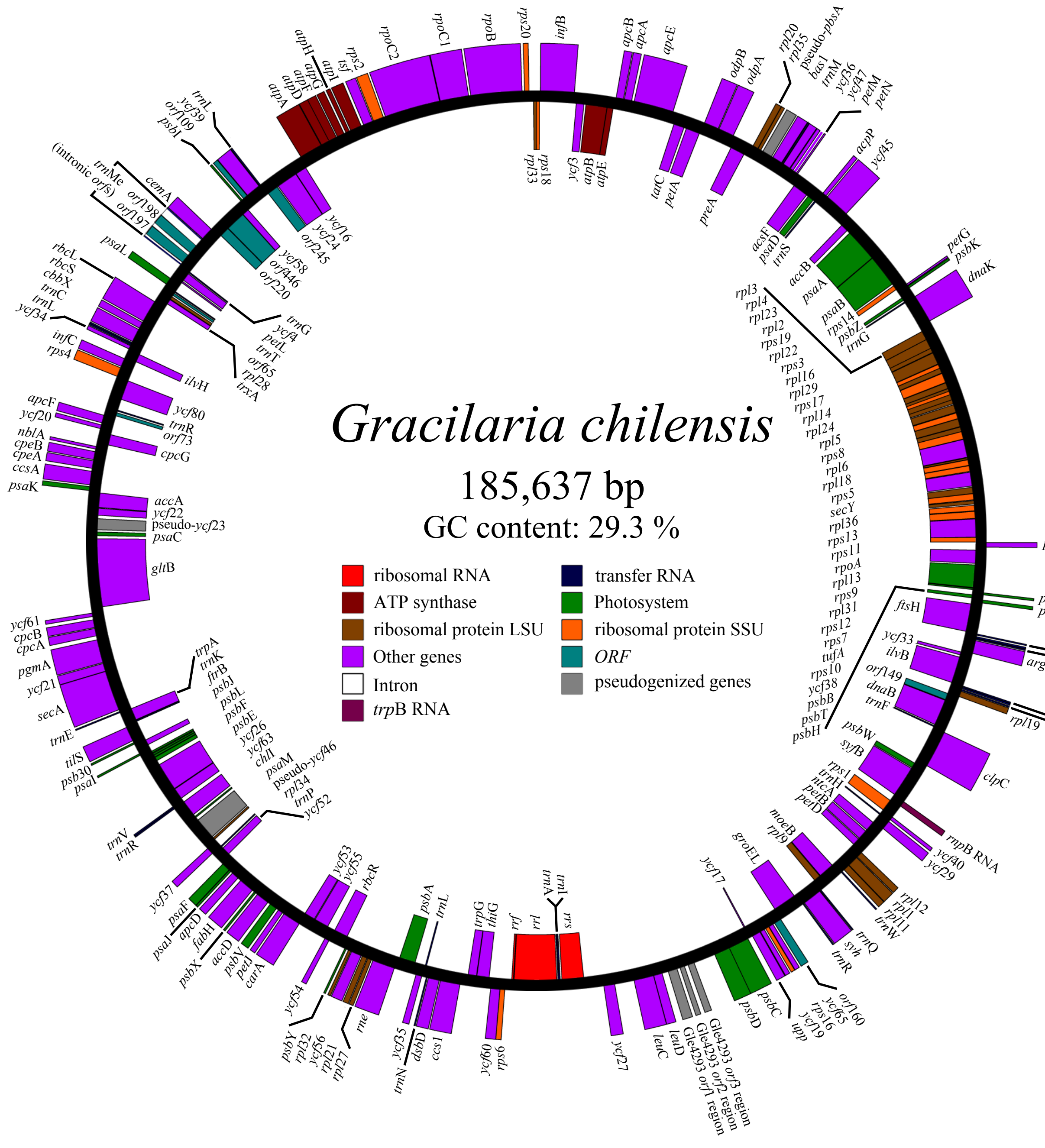

Fig. S5. Plastid genome map of *Gracilaria chilensis*. Functional gene groups are color-coded. Genes drawn inside of the circle indicate the transcriptionally clockwise strand, and genes on outside indicate the counter-clockwise strand.

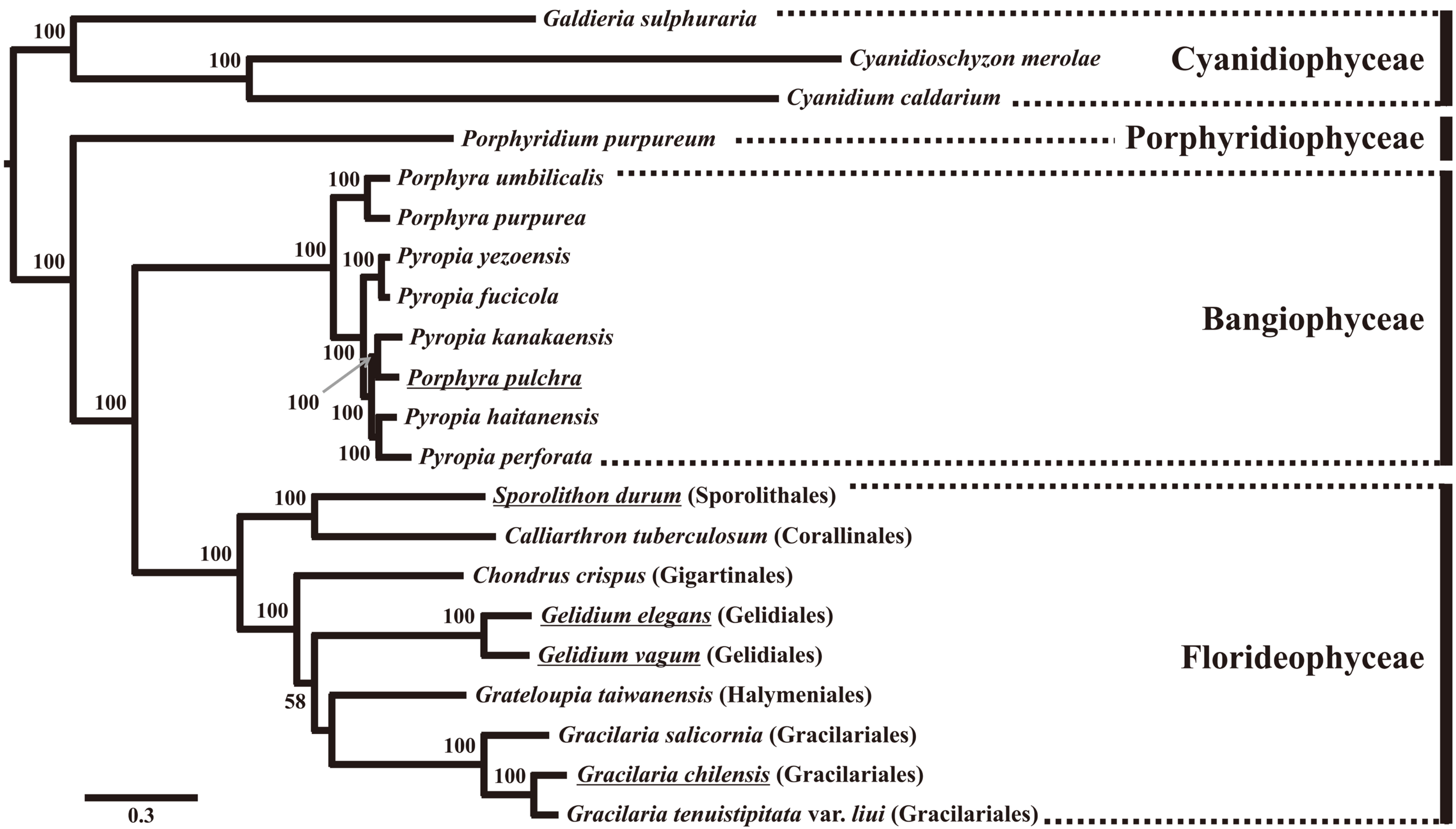

Fig. S6. Maximum likelihood tree based on aligned amino acid sequences of concatenated 193 protein encoding genes from 16 published and 5 new (underlined) plastid genomes (2000 replications, bootstrap support > 50%).

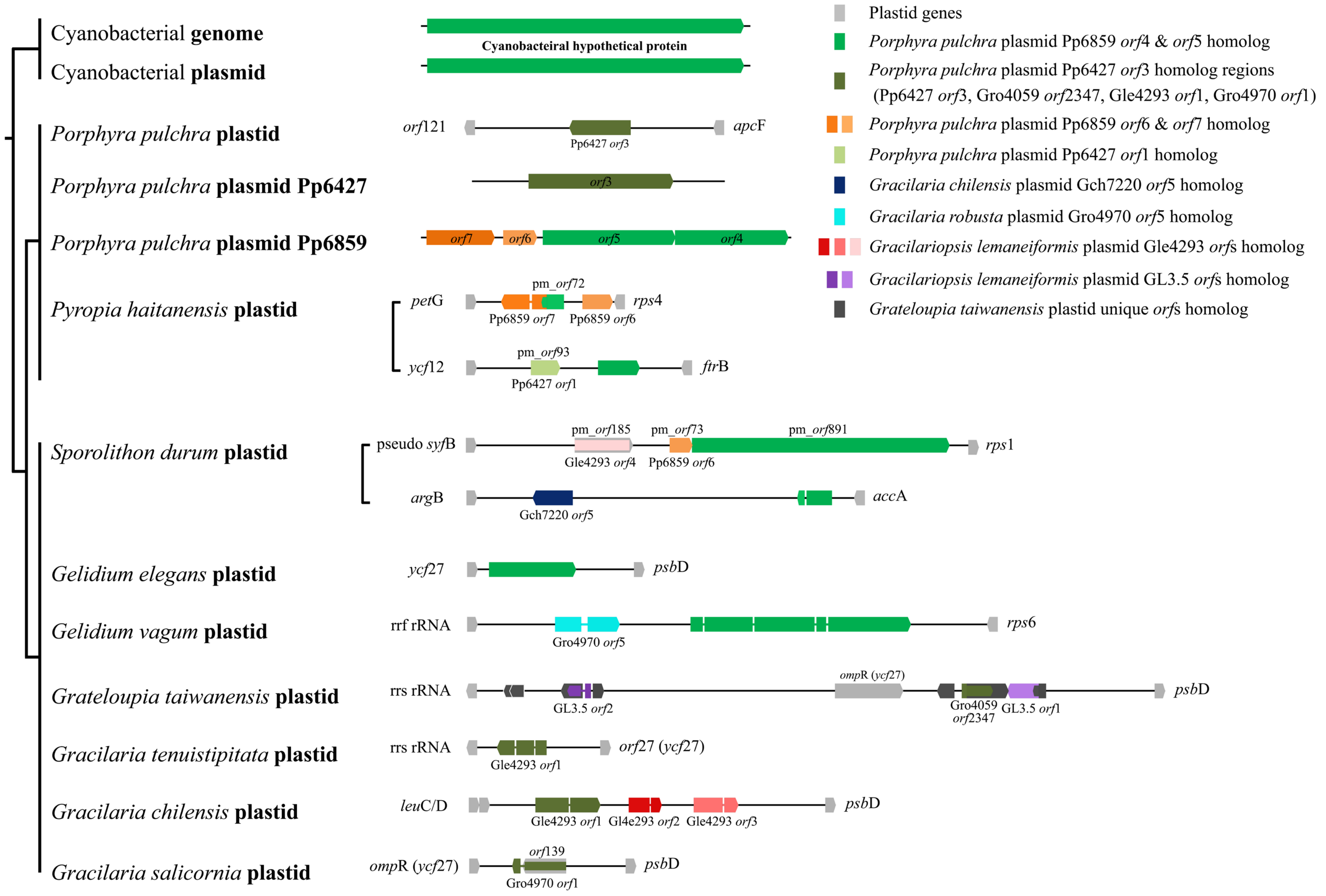

Fig. S7. Synteny of all plasmid-derived regions in red algae. Colored boxes indicate each homolog region in red algal plasmid and plastid genomes. *Porphyra pulchra* plasmid Pp6859 *orf*4 and *orf*5 homolog regions are also presented in some cyanobacterial species.

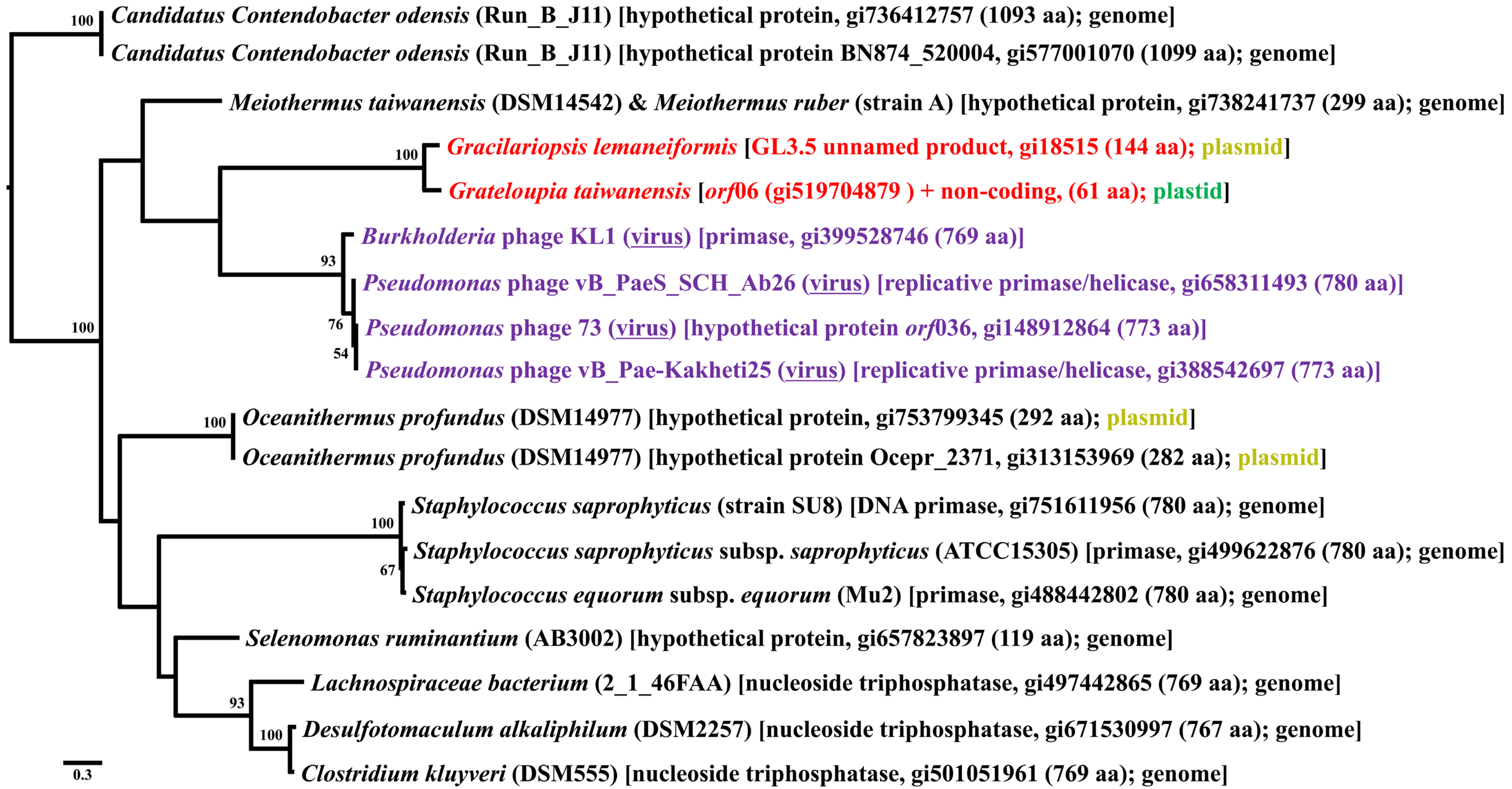

Fig. S8. Maximum likelihood tree based on aligned amino acid sequences of *Gracilariopsis lemaneiformis* plasmid GL3.5 *orf*2 related genes and its homologs from BLASTp search (cutoff e-value 1.0e-03) (2000 replications, bootstrap support > 50%). Taxon information and their sequence localities are color-coded as follows: bacteria (black), red algae (red), virus (violet), plastid (green) and plasmid (brown).

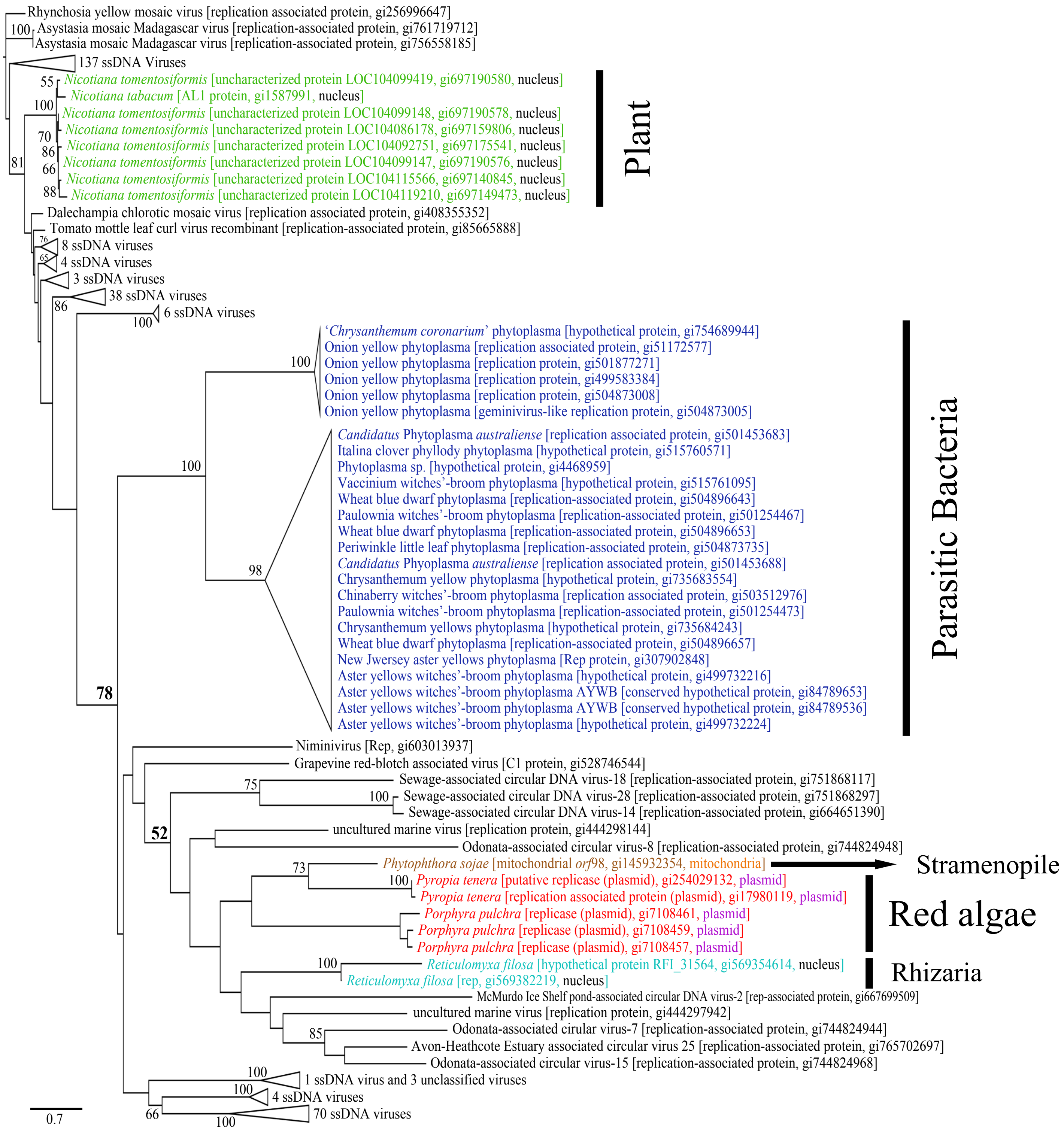

Fig. S9. Maximum likelihood tree based on aligned amino acid sequences of red algal replicase genes from *Pyropia tenera* (GI: 254029132, 17980119) plasmids, *Porphyra pulchra* plasmid (GI: 7108461, 7108459, 7108457) and their homologs from BLASTp search (cutoff e-value 1.0e-05) (2000 replications, bootstrap support > 50%). Taxon information and their sequence localities were indicated with color-code as follows: parasitic bacteria (blue), plant (green), red algae (red), rhizaria (cyan), stramenopile (brown), plasmid (violet), mitochondria (orange) and nucleus (black).
